# Improved drought stress tolerance in Arabidopsis by CRISPR/dCas9 fusion with a Histone AcetylTransferase

**DOI:** 10.1038/s41598-019-44571-y

**Published:** 2019-05-30

**Authors:** Joaquin Felipe Roca Paixão, François-Xavier Gillet, Thuanne Pires Ribeiro, Caroline Bournaud, Isabela Tristan Lourenço-Tessutti, Daniel D. Noriega, Bruno Paes de Melo, Janice de Almeida-Engler, Maria Fatima Grossi-de-Sa

**Affiliations:** 1 Embrapa Genetic Resources and Biotechnology, Brasília, DF Brazil; 2 INRA, Université Côte d’Azur, CNRS, ISA, Sophia Antipolis, France; 3 Catholic University of Brasilia - Post-Graduation Program in Genomic Sciences and Biotechnology, Brasília, DF Brazil

**Keywords:** Molecular engineering in plants, Chromatin remodelling

## Abstract

Drought episodes decrease plant growth and productivity, which in turn cause high economic losses. Plants naturally sense and respond to water stress by activating specific signalling pathways leading to physiological and developmental adaptations. Genetically engineering genes that belong to these pathways might improve the drought tolerance of plants. The abscisic acid (ABA)-responsive element binding protein 1/ABRE binding factor (AREB1/ABF2) is a key positive regulator of the drought stress response. We investigated whether the CRISPR activation (CRISPRa) system that targets AREB1 might contribute to improve drought stress tolerance in Arabidopsis. Arabidopsis histone acetyltransferase 1 (AtHAT1) promotes gene expression activation by switching chromatin to a relaxed state. Stable transgenic plants expressing chimeric dCas9^HAT^ were first generated. Then, we showed that the CRISPRa dCas9^HAT^ mechanism increased the promoter activity controlling the β-glucuronidase (*GUS*) reporter gene. To activate the endogenous promoter of *AREB1*, the CRISPRa dCas9^HAT^ system was set up, and resultant plants showed a dwarf phenotype. Our qRT-PCR experiments indicated that both *AREB1* and *RD29A*, a gene positively regulated by AREB1, exhibited higher gene expression than the control plants. The plants generated here showed higher chlorophyll content and faster stomatal aperture under water deficit, in addition to a better survival rate after drought stress. Altogether, we report that CRISPRa dCas9^HAT^ is a valuable biotechnological tool to improve drought stress tolerance through the positive regulation of AREB1.

## Introduction

Improving agronomic traits that give plants resistance to biotic and abiotic stresses to increase their economic value is a recurrent concern worldwide. The awareness of climate change and global warming emphasizes the need to implement some efficient and sustainable solutions. In several countries, a current important issue is the maintenance of crop production during drought^1–3^. Drought varies spatially and temporally as well as in strength. Accordingly, plants have diversified their responses and have evolved to exhibit multiple morphological and physiological behaviours^4,5^. These behaviors consist of different degrees of drought escape, avoidance and tolerance. Exploiting genetic traits that enhance the drought stress response while maintaining high yields remains of critical interest for crop management. Conventional breeding and transgenic approaches have been shown to improve drought stress tolerance in plants such as maize, soybean, rice and wheat^6,7^.

Comprehensive molecular analyses have deciphered the cellular pathways that orchestrate the drought stress response^8,9^. Abscisic acid (ABA) is a phytohormone that plays a role as a key regulator of the drought stress response in plants by regulating gene expression and controlling stomatal closure to prevent water losses by transpiration^10^. The basic leucin zipper (bZIP) transcription factors, termed ABA-responsive element binding proteins/ABRE binding factors (AREB/ABFs), are important determinants in ABA signalling^8^. Over-expression of *AREB1* (also named *ABF2*) exhibited enhanced drought stress tolerance in Arabidopsis, rice and soybean, while *AREB1* loss of function causes drought stress sensitivity^11–15^. Indeed, AREB1 regulates a large set of genes downstream of the ABA signalling pathway in response to drought stress^13^ and participates in osmotic stress protection, ABA biosynthesis and antioxidant signalling^12,16^. Thus, *AREB1* represents an attractive candidate gene for improving the drought stress response.

Targeted plant genome editing using CRISPR nucleases has become a promising approach to create new plant varieties^17–19^. Beyond genome editing, the CRISPR mechanism has been remodeled to accomplish CRISPR activation (CRISPRa) and CRISPR interference (CRISPRi)^20^. The catalytically inactive form of Cas9 (dead Cas9, abbreviated dCas9) has been fused with transcription activators and inhibitors to modify transcription through specific gene promoters and with chromatin modulator domains to facilitate targeted epigenome editing^21–23^. Histone acetyltransferase (HAT) catalyzes the acetylation of core histones through the addition of an acetyl group to the lysine residue on the terminal tail of the histones^24^. Histone acetylation triggers DNA relaxation and leads to exposure of DNA to the transcriptional machinery^25^. Thus, HAT activity is correlated with gene expression activation. In this way, the use of dCas9 in fusion with HAT (dCas9^HAT^), combined with the directed targeting of sgRNAs, appears promising for positively regulating the activity of a targeted promoter^26,27^. CRISPR/dCas9 epigenome editing of specific drought stress response genes therefore emerges as an encouraging strategy for improving stress tolerance in plants.

Herein, we asked whether an engineered dCas9^HAT^ could efficiently enhance *AREB1* gene expression in *Arabidopsis thaliana* in response to drought stress. We first generated stable Arabidopsis transgenic lines expressing dCas9 fused with the core catalytic domain of an Arabidopsis HAT. Next, we validated our CRISPR system using sgRNA targeting a β-glucuronidase (GUS) reporter system. Finally, we transformed dCas9^HAT^ lines with a construct containing sgRNAs targeting an *AtAREB1* promoter region and observed, by molecular and physiological approaches, an enhanced response to drought stress in these transgenic plants.

## Results and Discussion

### Generation of Arabidopsis transgenic lines expressing dCas9^HAT^

**Figure 1:**
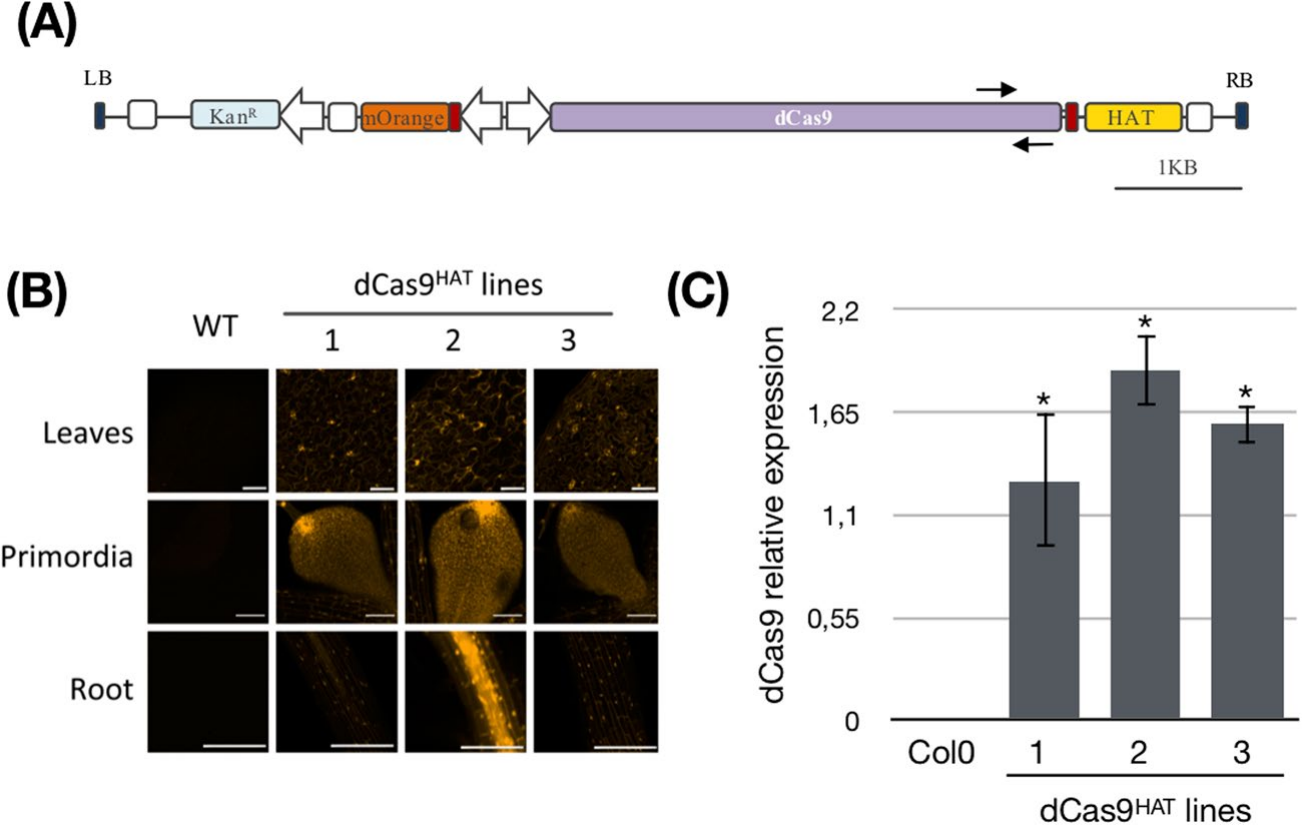
Molecular characterization of transgenic A. thaliana dCas9^HAT^ lines. (**A**) Schematic representation of the construct allowing the selection of A. thaliana dCas9^HAT^ lines. KanR: the kanamycin resistance gene; mOFP: monomeric orange fluorescent protein; NLS: nuclear localization signal. The white arrows indicate the cauliflower mosaic virus (CaMV) 35 S promoter; the white squares indicate the CaMV poly(A) signal (terminator); the black arrows indicate qPCR oligos (**B**) Fluorescence microscopy imaging of *A. thaliana* leaves, primordia and roots expressing the mOFP in the nucleus in three dCas9HAT lines compared with Col-0 plants. All confocal images were acquired under identical parameters (excitation: 549 nm/emission: 565 nm). Scale bars in the left inferior corner = 50 μm (**C**) Assessment of dCas9^HAT^ expression. RT-qPCR was performed in Col-0 plants and in three lines of dCas9^HAT^-transformed plants. Transcript levels were normalized against the geometric mean of the transcript levels of the housekeeping genes (GAPDH and Actin2). The mean and standard deviation (SD) were calculated from three independent biological replicates. The calibrator was chosen as the sample with the lowest expression of the transgene (excluding Col-0 plants). Asterisks indicate significant differences between Col-0 plants and the different lines (Wilcoxon test, *P < 0.05).

We first designed the dCas9^HAT^ construct to assay the transcriptional regulation of the gene of interest. The catalytic core from the Arabidopsis *Histone AcetylTransferase* *1* gene (*AtHAC1*, AT1G79000), was fused to the N-terminal part of dCas9 and cloned into a modified version of the plant binary vector pGreenII (Fig. 1A, Table S1). The T-DNA was designed to perform two rounds of plant transgenic selection based on antibiotic (kanamycin) resistance and the level of a fluorescence reporter gene (a nuclear mOrange fluorescent protein, mOFP). Once positive transformants were selected, fluorescence intensity was assessed by confocal microscopy (Fig. 1B). The fluorescence corresponding to mOFP could be seen all over the roots and leaves and was present at the highest intensity in the nuclei. Some transgenic lines displayed stronger fluorescence, suggesting that the cassette was inserted in an actively expressed region of the genome. Among the lines, the occurrence of the dCas9 construct was checked by PCR (Fig. S1), and gene expression was analyzed by qPCR (Fig. 1C). The transgenic line dCas9^HAT^ number 2 had the highest mOFP and dCas9 expression and was retained for this study (hereafter named dCas9^HAT^).

### Challenge of the dCas9 constructs in a GUS reporter system

**Figure 2:**
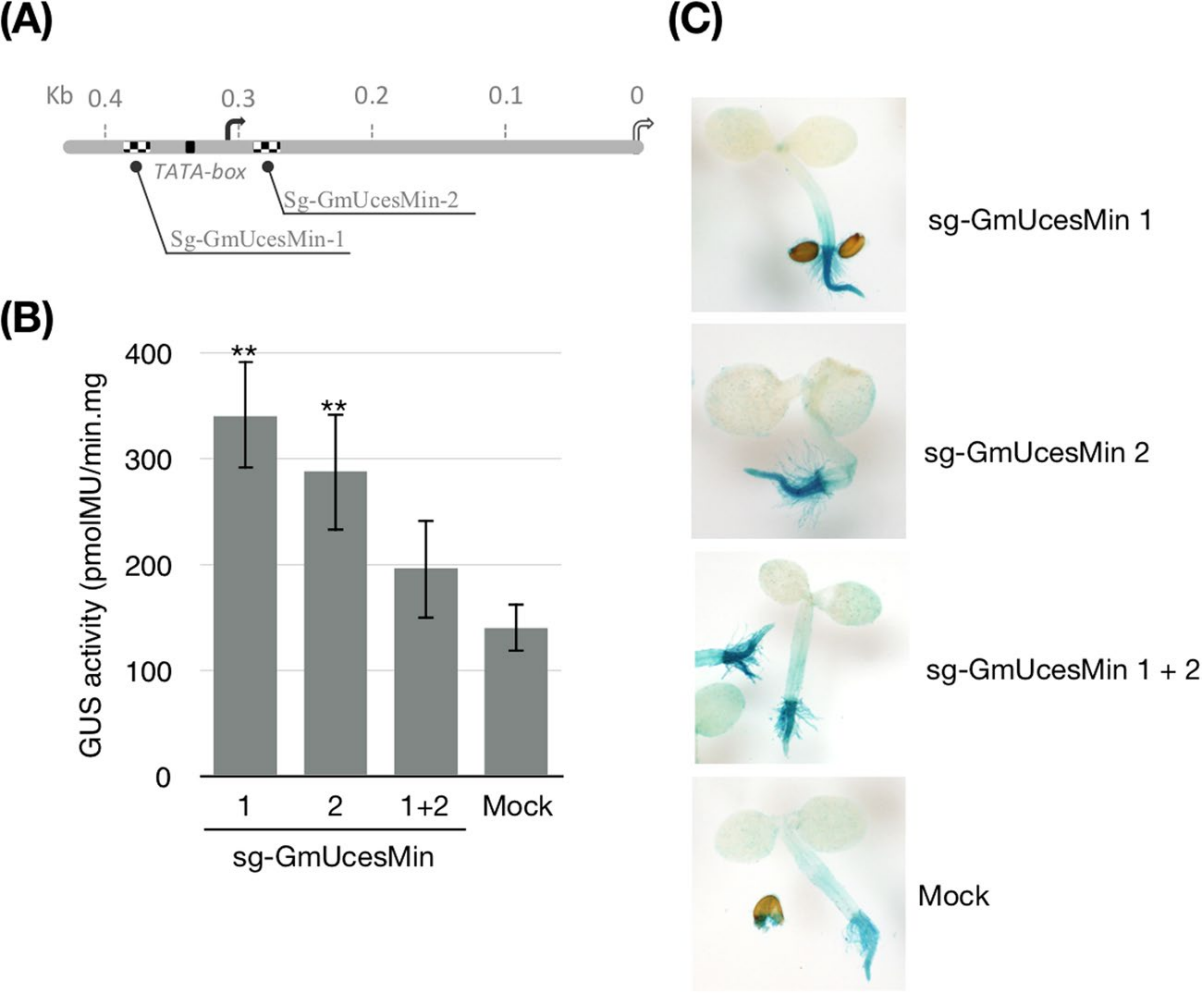
Challenge of dCas9^HAT^ in a GUS reporter system. (**A**) Schematic representation of the GmUcesMin promoter with the sgRNA positions (black and white squares). The TATA box is represented by a black square. The black and white curved arrows represent the TSS and ATG codon, respectively. (**B**) Arabidopsis seedlings from stably transformed dCas9^HAT^ fusions were transiently transformed with GmUcesMin and different combinations of sgRNAs. The results are presented as the mean and SD of 6 independent experiments (n = 20 pooled seedlings per experiment). Asterisks indicate significant difference between the GUS activity for each dCas9 fusion guided by one sgRNA or a combination of two sgRNAs compared to mock controls (Student’s t-test, *P < 0.05). Bars indicate the standard error. (**C**) GUS staining was performed for the GmUcesMin promoter in the same transiently transformed seedlings.

To evaluate the dCas9^HAT^ construct, we set up a surrogate reporter system based on regulation of GUS reporter gene expression. We used the 170-bp minimal truncated version of the *Glycine max* ubiquitin promoter, herein designated GmUcesMin^28,29^. We selected two sgRNAs near the transcription start site (TSS) of GmUcesMin (Fig. 2A, Table S2). The efficiency of dCas9^HAT^ in activating the GUS reporter system was quantified by its enzymatic activity. Seedlings of stably transformed Arabidopsis lines expressing dCas9^HAT^ were incubated with *Agrobacterium* carrying Ti plasmids to perform transient ectopic expression of GmUcesMin-GUS in combination with the expression of one or two sgRNAs. Significantly elevated enzymatic activity was observed for sgRNA1 (~2.4-fold increase) and sgRNA2 (~2-fold increase), while enzymatic activity was elevated ~1.4-fold for the combination of the two sgRNAs. This result indicates that the expression of dCas9^HAT^ enhanced the expression of the GUS gene in *trans* when targeted to GmUcesMin promoter. Remarkably, some substantial differences were noted depending on the location of the sgRNA and/or the sgRNA combination. Previous studies have suggested that the distance of the sgRNA from the TSS might influence the transcriptional regulation of the gene of interest. While some studies have reported that a specific sgRNA binding distance from the TSS (−50 bp to +300 bp) corresponds to higher target gene expression^30,31^, others have pointed out that dCas9 might generate steric hindrance and thus interfere with transcriptional machinery activities^21,32^. Regarding the construct GmUcesMin, our two sgRNAs are separated only by 118 bp. Considering the 3D conformation of the DNA, the 30 bp length occupied by the dCas9 on DNA^33^ and the space taken by the HAT domain, a steric hindrance effect could be responsible for destabilizing locally the protein complexes standing on DNA. Compared to the use of only one sgRNA, this collateral effect might result to lower gene expression rather than improve it.

We determined the expression profile of GmUcesMin-GUS by GUS staining (Fig. 2C). Since dCas9^HAT^ and the sgRNA are supposed to be broadly co-expressed in the whole Arabidopsis plant, we expected to observe GUS activity in every tissue. However, GUS activity was restricted to the roots and was higher in transgenic plants than in controls. We hypothesized that some root-specific transcription activators might trigger GUS expression specifically in roots. However, the mapping of different regulatory boxes in GmUcesMin identified several regulatory elements but did not discriminate whether they are specific to the root (Fig. S2). The transgenic plants differed from the controls only in intensity. These results suggest that dCas9^HAT^ in combination with sgRNA enhances gene expression in the roots but does not activate gene expression in other tissues. Notably, Zhang *et al*.^29^ previously reported that the full-length GmUcesMin (called GmScreamM2, length: 1391 bp) triggers gene expression principally in soybean seeds. It is tempting to postulate that the truncated form of GmUcesMin and/or our expression conditions might favor the expression of the target gene in roots.

### Molecular and phenotypic characterization of dCas9^HAT^-sgA

**Figure 3:**
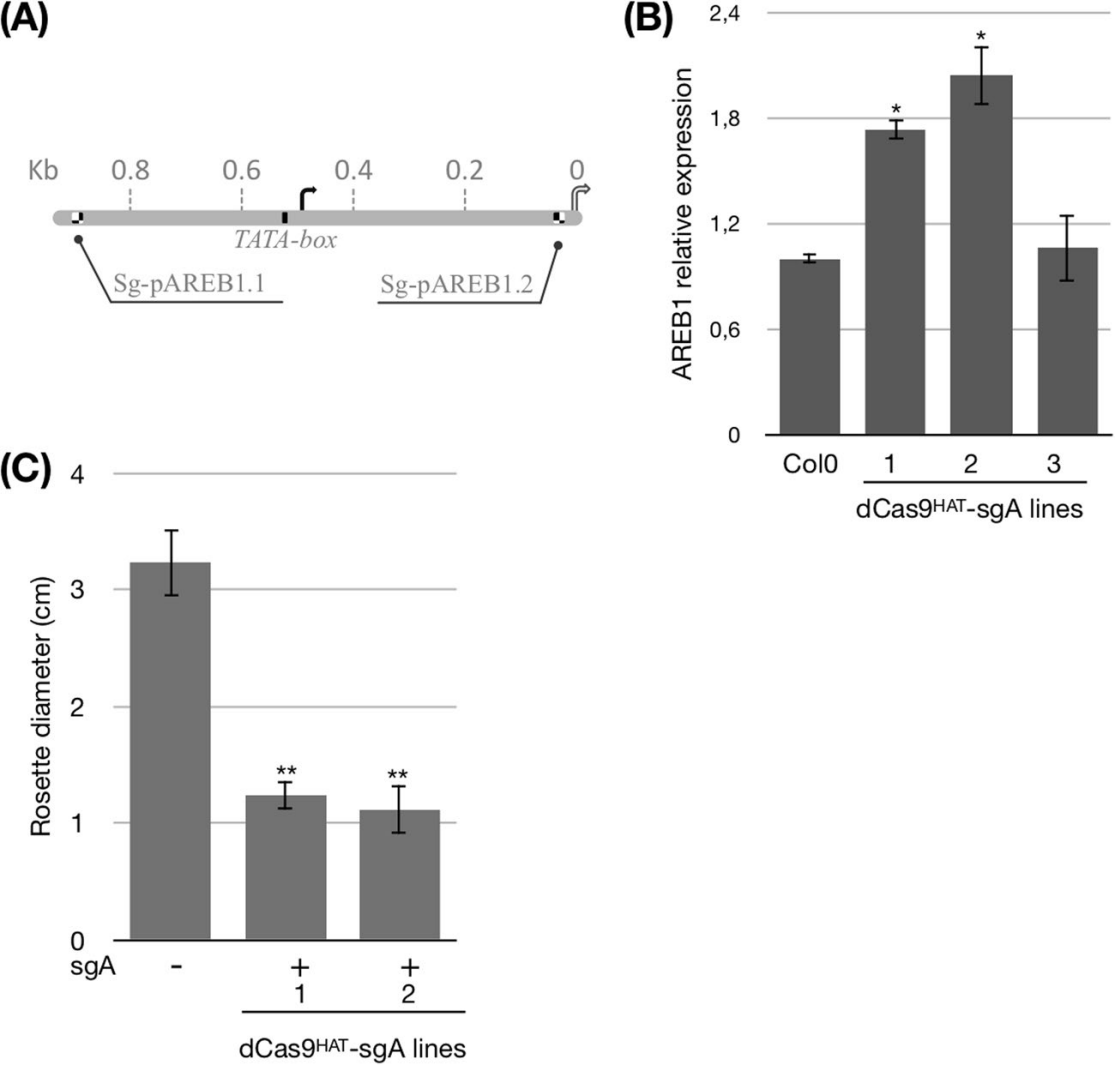
Challenge of dCas9^HAT^ constructs in the transcriptional regulation of *AREB1* by targeting p*AREB1*. (**A**) Schematic representation of p*AREB1* with the two sgRNAs designated. The TATA box is represented by a black square. (**B**) Relative expression of the *AREB1* and (**C**) Phenotypic analysis of dCas9^HAT^-sgA. Rosette diameter of three-week-old plants. The results represent the mean of n = 13. dCas9^HAT^ control plants are represented by a – symbol, and dCas9^HAT-^sgA2 plants are represented by a + symbol. Asterisks indicate significant difference between dCas9-sgA lines and dCas9 control lines (Wilcoxon test, *P < 0.05). Bars indicate standard error.

We next inquired whether *AREB1* gene expression could be regulated by dCas9^HAT^. We designed two sgRNAs to target the endogenous promoter of *AtAREB1* (Fig. 3A). One sgRNA is located at 3′ from the TSS (−479 bp), and the second is in the 5′ UTR (+356 bp). The two sgRNAs (sg-pAREB1.1 and sg-pAREB1.2) were cloned in tandem within a single T-DNA and transformed into the Arabidopsis dCas9^HAT^ transgenic lines to generate dCas9^HAT^-sgA. We verified *AREB1* gene expression in three transgenic lines by real-time qPCR (Fig. 3B). In each experiment, the control line was the parental line, dCas9^HAT^. We observed a slight but significant 1.7-fold increase in *AREB1* expression in the dCas9^HAT^-sgA1 line and a 2-fold increase in the dCas9^HAT^-sgA2 line compared to that in the control line, suggesting that targeting dCas9^HAT^ to the *AREB1* gene could trigger its transcription. Three weeks after germination, the rosette diameter in the dCas9^HAT^-sgA2 and dCas9^HAT^-sgA1 plants was ~3-fold smaller than in the controls (Figs 3C, S3A,B). The leaf length was also smaller for both lines, respectively, than in the control line (Fig. S3C). These results suggest that the mutant caused a dwarf phenotype under normal plant growth conditions. They also corroborate phenotypic traits related to drought stress shown by Fujita *et al*.^12^, in which the over-expression of the constitutive form of AREB1, AREB1ΔQT, presented smaller phenotypes and the *areb1* mutants had larger rosettes. Interestingly, we observed that without water deficit, *AREB1* is slightly positively regulated, indicating that dCas9^HAT^ activates *AREB1* apart of the context of drought.

The activation of promoters via histone H3K27 acetylation through the use of dCas9^p300Core^ domains has been evaluated in animal cells, and the dCas9^p300Core^ domains have been shown to be more potent than activation domains^21,34,35^. Further studies have also reported that the over-expression of histone deacetylases (HDACs) plays a role in the ABA response and that the use of an HDAC inhibitor induces hyperacetylation and increases *AREB1* gene expression in peanut^36,37^. Altogether, our findings suggest that histone acetylation by dCas9^HAT^ at *AREB1* loci is a determinant parameter in *AREB1* expression and allows local chromatin rearrangement.

### dCas9^HAT^-sgA plants have enhanced drought stress tolerance

**Figure 4:**
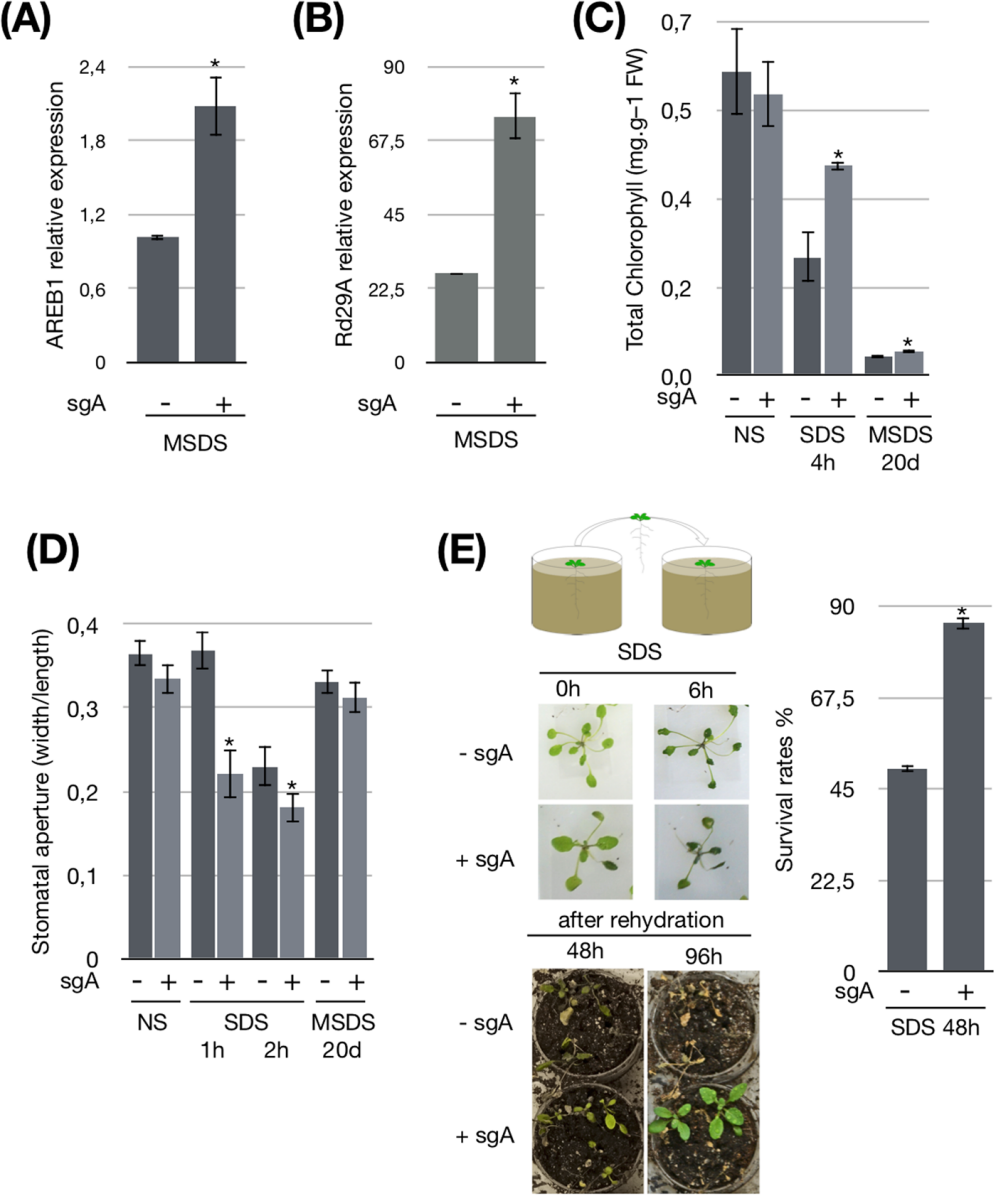
Molecular and physiological analyses of drought stress responses in dCas9^HAT-^sgA. Transcript levels of (**A**) *AREB1* and (**B**) *RD29A* in dCas9^HAT^ and dCas9^HAT^-sgA2 plants during drought stress. Expression levels were normalized against the geometric mean of the expression of the housekeeping genes (GAPDH and Actin2). The mean and SD were obtained from three biological replicates. Asterisks indicate significant differences between the control and transformed plants (Wilcoxon test, *P < 0.05). For each gene, the expression level in the dCas9^HAT^ control was defined as the calibrator (1.0). (**C**) Total chlorophyll content in non-stressed plants 4 h after SDS and after MSDS. The results represent the mean of n = 6. (**D**) Stomatal aperture measurements after 2 h and 4 h of severe stress and 20 days of drought stress. The results are presented as the mean of n = 30. Asterisks indicate significant difference between co-transformed plants and control lines (Student’s t-test, *P < 0.05). The bars indicate standard error. (**E**) Survival rates after 6 h of SDS and 48 h and 96 h of rehydration. The results represent the percentage of surviving plants (n = 20). An asterisk indicates a significant difference between the control dCas9^HAT^ and dCas9^HAT^-sgA2 plants (chi-square test, *P < 0.05). dCas9^HAT^ control plants are represented by a – symbol, and dCas9^HAT^sgA2 plants are represented by a + symbol.

We next analyzed the efficiency of the dCas9^HAT^ activator at a molecular level by measuring the gene expression of *AREB1* and *RD29A*, a gene positively regulated by AREB1 and a reporter gene for AREB1 activity under drought stress^12,38^. The expression levels of *AREB1* and *RD29A* were significantly increased by ~2-fold and ~3-fold respectively in dCas9^HAT^-sgA2 plants (Fig. 4A,B), and ~2-fold and ~2.6-fold in dCas9^HAT^-sgA1 plants (Fig. S4). These data suggest that the drought stress response is enhanced in the dCas9^HAT^-sgA lines. We next investigated the physiological traits of transgenic Arabidopsis seedlings during drought. Only the dCas9^HAT^-sgA2 was selected for further physiological analyses. We performed this investigation under two drought conditions. Severe drought stress (SDS) was produced by removing plants from the soil and keeping them at 20% humidity on plates on the laboratory bench. In another experiment, mild-severe drought stress (MSDS) was produced through water withdrawal for up to 20 days. The photosynthesis is affected by drought stress, and a decrease of chlorophyll content is an indicator of photosynthesis activity^39,40^. We solvent-extracted the chlorophyll from leaves and subsequently measured the chlorophyll fluorescence by UV-visible spectroscopy^41^. When plants were normally irrigated, the content of chlorophyll was comparable between the dCas9^HAT^-sgA2 line and the control line (Fig. 4C). In contrast, 4 h after SDS, the content of total chlorophyll was 1.7-fold higher in dCas9^HAT^-sgA2 plants. After twenty days of MSDS, dCas9^HAT^-sgA2 plants had significantly higher content of chlorophyll (1.3-fold) than the control plants. Our findings likely indicate that the higher chlorophyll content found in the dCas9^HAT^-sgA2 line is in agreement with an improved drought stress response at a physiological level.

Another characteristic of the drought stress response is a decrease in stomatal aperture to limit water loss^42^. We measured stomatal width and length by confocal imaging and calculated the stomatal aperture (width/length) in non-stressed versus stressed plants after SDS (1 h, 2 h) and MSDS. In dCas9^HAT^-sgA2 plants without drought stress, the stomatal aperture was comparable to that of the control plants. However, 1 h after drought stress, the stomatal aperture was 1.6-fold lower in dCas9^HAT^-sgA2 plants than in control plants (Fig. 4D). After 2 h, the stomatal aperture was still significantly lower in dCas9^HAT^-sgA2 plants, even though control plants also partially closed their stomata between 1 h and 2 h of stress. However, our data revealed that stomatal closure was triggered more rapidly in dCas9^HAT^-sgA plants. After 20 days of stress, the stomatal aperture of the dCas9^HAT^-sgA plants was comparable to that of the control plants, suggesting that dCas9^HAT^-sgA2 does not regulate stomatal aperture differently during long-term drought stress. Our results indicate that the expression of dCas9^HAT^-sgA2 leads to faster stomatal closure after severe drought stress and corroborate a previous study stating that AREB1 might be partially associated with stomatal closure^13^.

Finally, we performed a survival assay to analyze the ability of transformed plants to recover after SDS and MSDS (Figs 4E; S5). After SDS followed by 48 h of rehydration, we observed that 85% of the dCas9^HAT^-sgA2 plants showed significant total recovery, whereas only 50% of the control plants were still alive. However, after 96 h of rehydration, pictures showed that transgenic plants present a recovered vigor compared to control plants (Fig. 4E). When we subjected the plants to MSDS and then rehydration for 48 h, we observed that all the transgenic plants survived, whereas all the controls died (Fig. S5). Altogether, our data indicate that dCas9^HAT^ directed to *AREB1* loci improves the Arabidopsis drought response.

## Conclusion

**Figure 5:**
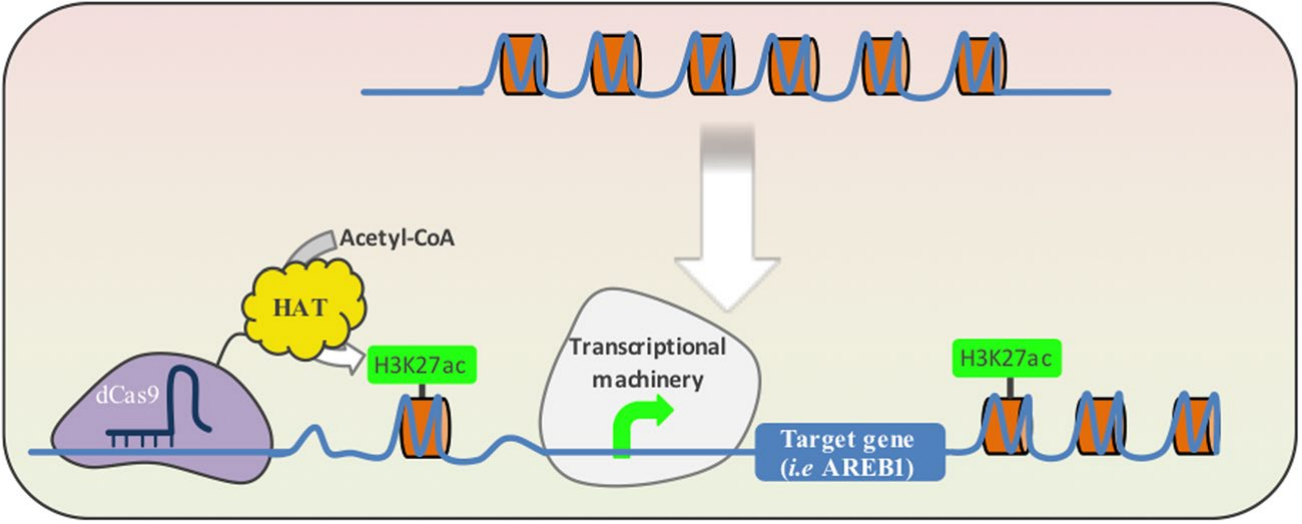
Schematic illustration of a model for dCas9^HAT^ function in transcriptional activation of a target gene. Upper: the histone compaction (in orange) induces DNA condensation and limits transcription. Below: the dCas9HAT in complex with a single guide RNA (in dark blue) binds DNA on a target locus. The histone acetyl-transferase (HAT) triggers histone acetylation on the lysine 27 (H3K27ac) and induces local DNA relaxation. The DNA relaxation strengthens the interaction of the transcriptional machinery and /or transcriptional enhancers with the target locus.

In addition to the use of modulators to aid the recruitment of RNA polymerase (Pol II) transcription machinery, the use of domains that modify chromatin folding is another interesting way to fine-tune gene expression. The expression of dCas9^HAT^ allows acetylation of lysine 27 of Histone 3 (H3K27ac) favoring the unwind of chromatin and enhancing the interaction with transcriptional enhancers as the assembly of the transcriptional machinery (Fig. 5). This approach has been demonstrated in animals. In the present study, our main finding was that dCas9^HAT^ positively regulates *AREB1* and produces an enhanced drought stress response. It is noteworthy that dCas9^HAT^ activity depends on the cellular context. The enhancer effect of dCas9^HAT^ is stronger when the drought stress response is activated. This finding suggests that the chromatin folding at the *AREB1* locus constitutes a regulatory mechanism for *AREB1* gene expression. We also report that GUS expression varies based on number of sgRNAs used and their respective positions. Having a better understanding of the chromatin context of a specific locus will help in the rational design of CRISPRi/a strategies.

## Experimental procedures

### Plasmid construction and sgRNA design

The sequence encoding the *A. thaliana* acetyltransferase domain was gathered from TAIR from the protein HAC1 (AT1G79000, between amino acids (AA) 1119 and 1408), synthesized (EPOCH) and cloned into a vector containing the sequence encoding dCas9 (pAWG-dCas9-VPR, which was a gift from George Church; plasmid #63802; http://n2t.net/addgene:63802; RRID: Addgene_63802)^43^. The cassette containing dCAS9^HAT^ was cloned into a pGreen KII (pGKII_dCas9-VPR) vector containing a NeoR/KanR selection region and a sequence coding for the orange fluorescent protein (OFP). The sgRNAs were designed and screened using CHOPCHOP (http://chopchop.cbu.uib.no). The *in-silico* analysis of cis-acting elementos in UcesMin promoter was also considered for the design of sgRNAs 1, 2 and 3 (Fig. S2). The promoter sequence of *AREB1* (AT1G45249.3) was extracted from the SeqViewer tool on the TAIR website, and sgRNAs targeting the *AREB1* promoter (sg-pAREB1.1 and sg-pAREB1.2) were designed at −479 bp and +356 bp from the predicted TSS (approximately 25 bp downstream from the TATA box). A second vector was designed containing a sgRNA scaffold and two cassettes for sgRNA expression, with sg-pAREB1.1 and sg-pAREB1.2 controlled by the U6 promoter. Box sequences called Gibson boxes^44^ were designed at the edges and the middle of each sgRNA template to facilitate amplification and cloning. sgRNAs targeting the GmUcesMin promoter were chosen to have two different locations around the TSS. The sgRNA templates were amplified in a three-step PCR with one pair of primers in the Gibson boxes (extremities) and a second pair spanning the new 20-nt sgRNA and 20 nt in the template (Table S3). Each sgRNA template was sub-cloned into *SmaI*-linearized pGKII0229.

### Plant material, growth conditions and stable transformation

Col-0 *A. thaliana* seeds were surface-sterilized and germinated *in vitro* in Murashige and Skoog (MS) medium including vitamins (with or without selection agents). After stratification (2 days at 4 °C), the seeds were germinated and grown under a 12-h photoperiod in a growth chamber at 21 °C. Alternatively, plants were sown on a soil/sand mixture, stratified and grown under a 16 h photoperiod in a growth chamber at 21 °C. The constructs were transferred into *Agrobacterium tumefaciens* GV3101 via heat-shock (30 minutes at 0 °C, 5 minutes at 37 °C and 2 h at 28 °C in Luria-Bertani (LB) medium)^45^. The floral dip method was used to transform the *A. thaliana* plants^46^.

### Transient expression in *A. thaliana* seedlings

Transient expression in Arabidopsis seedlings was adapted from the FAST and AGROBEST protocols^47,48^. Briefly, 20 seedlings were stratified and germinated in ½ MS in 96-well plates. After 4 days in a growth chamber (12 h photoperiod, 21 °C), seedlings were co-incubated with the *A. tumefaciens* strain GV3101 (OD600 = 0.2) carrying constructs to allow the expression of GmUcesMin-GUS, sg-UcesMin.1 and/or sg-UcesMin.2. The co-culture medium consisted of a combination of 50% ½ MS, 0.25% sucrose and 50% ABMES salt medium (17.2 mM K_2_HPO_4_, 8.3 mM NaH_2_PO_4_, 18.7 mM NH_4_Cl, 2 mM KCl, 1.25 mM MgSO_4_, 100 µM CaCl_2_, 10 µM FeSO_4_, 50 mM MES, 2% glucose, and 200 μM acetosyringone). To control the agrobacterium population, we used an identical ratio o *A. tumefasciens* with each construct corresponding to 1/3 of the total bacterial population. If only one sgRNA was used, the rest 1/3 was composed of *A. tumefasciens* expressing an empty vector. After two days of co-cultivation, a fraction of the seedlings (5 of 20) were washed in distilled water for GUS staining. The remaining seedlings were put on MS with carbenicillin (100 µg/mL) for 1 day and then collected for GUS activity assays.

### Promoter activity assays

β-Glucuronidase (GUS) histochemical staining was performed by adding X-Gluc solution (2 mM X-Gluc ((5-bromo-4-chloro-3-indolyl) β-D-glucuronic acid) in 50 mM Na_2_HPO_4_ (pH 7.0) and 0.1% Triton X-100) to *A. thaliana* seedlings for 10 h at 37 °C. Seedlings were washed in 70% ethanol overnight and observed using bright field optics with a digital camera (Olympus MVX10). Promoter activity was monitored by a fluorometric GUS enzymatic assay^49^. The total soluble protein was extracted with a GUS extraction buffer (50 mM sodium phosphate buffer, 10 mM EDTA, 0.1% sarkosyl, 0.1% Triton X-100 and 10 mM β-mercaptoethanol) and quantified using the Bradford method (Bradford, 1976). The assay was carried out by adding 20 µg oftotal soluble protein to 1 mM 4-MUG (4-methyl-umbelliferyl-glucuronide) fluorogenic substrate (excitation: 356 nm; emission: 455 nm) and sampling 200 µLinto a stop buffer (0.2 M sodium carbonate) at three different time points (0, 15 and 30 minutes). A standard dilution curve was created with different concentrations of 4-MU (4-methyl umbelliferone). The fluorescence was measured using a SpectraMax M3 fluorometer (Molecular Devices).

### *In silico* analysis of the GmUcesMin promoter

The presence of cis-acting elements in the GmUcesMin promoter was examined using the bioinformatics tool MatInspector version 8.0 (Genomatix®). The term “plants” was used as the matrix group, “0.85” was used as the value for the similarity of the main bases that constitute each cis-acting element (core similarity), and “Optimized +1” was used as the value for the matrix similarity^50^.

### Gene expression analysis by qPCR

RNA extraction from *Arabidopsis thaliana* leaves was performed with a Concert™ Plant RNA Reagent kit (Invitrogen) following the manufacturer’s protocol. For cDNA synthesis, the following reagents were added to a 0.2-mL tube: 1 µg of total RNA, 1 µL of 10 µM NV-dT30 (2 µM), 1 µL of 10 mM dNTP (2 mM), and Milli-Q water to a total volume of 12 µL. The reaction was then incubated at 65 °C for 5 minutes. Then, 4 µL of 5 × First-Strand Buffer with 0.1 M DTT and 1 µL of RNase Out were added, and the reaction was incubated at a 37 °C for 2 minutes. Finally, 1 µL of the MMLV enzyme was added to the reaction; the reaction was incubated for 50 minutes at 37 °C and then inactivated for 15 minutes at 70 °C. The cDNA obtained in the previous step was diluted 1:20 and analyzed in biological and experimental triplicates for the differential expression of the *AREB1*, *RD29A* and *dCAS9* genes by real-time PCR. The qPCR reaction was performed as follows: 2 µL of cDNA (1:20), 5 µL of 2 × GoTaq qPCR Master Mix SYBR Green, 0.5 µL of each primer and 2 µL of water to a final volume of 10 µL. Amplification was performed in a CFX96 machine (BioRad). GAPDH (AT1G16300) and Actin2 (AT3G18780) were used as reference genes for relative quantification with the 2^(ΔΔCt) method^51,52^.

### Drought stress and morphological and physiological analyses

Transgenic *A. thaliana* seeds were sown in pots containing similar proportions of soil and sand and were regularly watered. After one week, plants were transferred to pots containing 3 transgenic plants and 3 control plants. For mild to severe drought stress (MSDS), after approximately 3–4 weeks, the soil was water saturated, and the plants were then kept without water for 25 days. Subsequently, plants were rehydrated for survival rate measurements. Rosette radius measurements were made and leaf morphology was examined in three-week-old plants^53^ with the aid of the LeafJ plugin for ImageJ^54^. Severe drought stress (SDS) conditions were created by removing whole plants from pots or by cutting the rosettes and placing them on the bench for 1 h to 6 h. Whole-rosette dehydration during SDS treatment was determined by cutting rosettes from three-week-old plants and weighing them hourly. To determine survival rates, whole seedlings were removed from the soil without harming the roots, and SDS was applied for 6 h on the bench. Both transformed and control plants that presented root damage were eliminated. The plants that had survived after rehydration for 48 h were recorded. Stomatal aperture was assessed from stressed leaves collected after 2 h, 4 h and 20 days. Confocal images of stomata were acquired under identical settings and processed with a Zeiss LSM 880 confocal fluorescence microscope using the software package LSM 510 version 3.2. The width and length of the stomatal apertures were captured by confocal microscopy, and the width-to-length ratios were calculated. After 24 h of treatment, chlorophyll was extracted from leaves with 80% acetone, and the chlorophyll content was estimated. After centrifugation at 12.000 × *g* for 5 minutes, absorbance was measured with a spectrophotometer at wavelengths of 645 and 663 nm. The chlorophyll concentration was estimated following Arnon’s equations: (Chlorophyll *a* (μg/mL) = 12.7 (A663) − 2.69 (A645); Chlorophyll *b* (μg/mL) = 22.9 (A645) − 4.68 (A663); Total chlorophyll (μg/mL) = 20.2 (A645) + 8.02 (A663))^55^.

### Statistical analysis

At least three independent replicates were conducted for each determination. For gene expression, Wilcoxon test was used to compare Ct values. For Gus activity of Physiological tests, Student’s t-test was used to compare the means. A difference was statistically significant when P < 0.05. Error bars in figures represent standard deviation (SD) of the means.

## Supplementary information

Supplementary data

Supplementary tables

## Data Availability

The datasets generated during and/or analyzed during the current study are available from the corresponding author on reasonable request.
