# Supplementary material for: Improved drought stress tolerance in Arabidopsis by CRISPR/dCas9 fusion with a Histone AcetylTransferase
Source: Sci Rep. 2019 May 30;9:8080. doi: 10.1038/s41598-019-44571-y (PMC6542788; doi:10.1038/s41598-019-44571-y)
Supplement: Supplementary file 1 — Supplementary data [file 41598_2019_44571_MOESM1_ESM.pdf]

IMPROVED DROUGHT STRESS TOLERANCE IN ARABIDOPSIS BY CRISPR/DCAS9 FUSION WITH A HISTONE ACETYLTRANSFERASE

Joaquin F. Roca Paixão[1,2*§], François-Xavier Gillet [1*], Thuanne Pires Ribeiro[1], Caroline Bournaud[1], Isabela Tristan Lourenço-Tessutti[1], Daniel D. Noriega[1], Bruno Paes de Melo[1], Janice de Almeida Engler[2], Maria Fatima Grossi-de- Sa[1,3, §]

[1]Embrapa Genetic Resources and Biotechnology, Brasília – DF, Brazil.
[2] INRA, Université Côte d'Azur, CNRS, ISA, France.
[3]Catholic University of Brasilia - Post-Graduation Program in Genomic Sciences and Biotechnology, Brasília - DF, Brazil

[*]Authors contributed equally to the work.
[§]Corresponding authors: E-mail: joaquinfrp@gmail.com, fatima.grossi@gmail.com

SUPPLEMENTARY INFORMATION

Figure S1. Detection of positive dCas9^{HAT} Arabidopsis plants via PCR. Genomic DNA was extracted from the leaves of selected plants to amplify the region between the dCas9 sequence and the HAT domain sequence (1214 bp amplicon, CAs9 FWRD and HAC REV primers). C−, negative control; C+, positive control (the PCR template was the original dCas9^{HAT} expression cassette used for plant transformation; M, 1.0-kb ladder (Invitrogen Cat. # 10787018).

Figure S2. *In silico* analysis of the GmUcesMin promoter. The sequence of GmUcesMin was analyzed with the MatInspector tool, version 8.0 (Genomatix®), and regulatory boxes were schematized.

Figure S3. Dwarf phenotype of dCas9^{HAT}-sgA2 plants. (A) Image of 3-week-old dCas9^{HAT}-sgA1 and 2 plants. (B) Images of independent leaves of 3-week-old dCas9^{HAT}-sgA1 and 2 plants (C) Images of 2-week-old in vitro cultivation of dCas9^{HAT}-sgA1 and 2 plants.

Figure S4. Molecular analyses of drought stress responses in dCas9^{HAT-}sgA1. Transcript levels of (A) *AREB1* and (B) *RD29A* in dCas9^{HAT} and dCas9^{HAT}-sgA1 plants during drought stress. Expression levels were normalized against the geometric mean of the expression of the housekeeping genes (GAPDH and Actin2). The mean and SD were obtained from three biological replicates. Asterisks indicate significant differences between the control and transformed plants (Wilcoxon test, $^{**}P<0.01$). For each gene, the expression level in the dCas9^{HAT} control was defined as the calibrator (1.0).

Figure S5. Images of dCas9^{HAT}-sgA2 and dCas9^{HAT} control plants subjected to MSDS and rehydrated for 48 h.

Figure S1

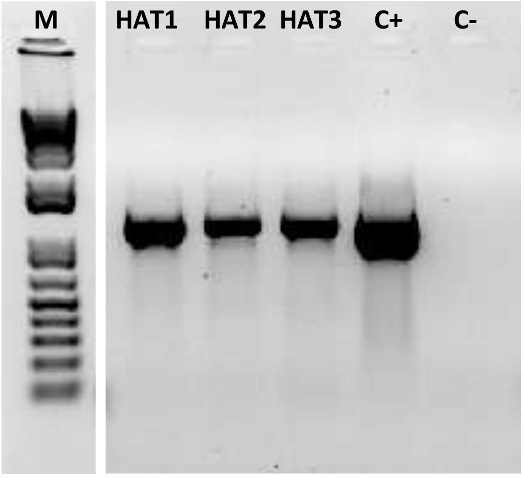

Figure S2

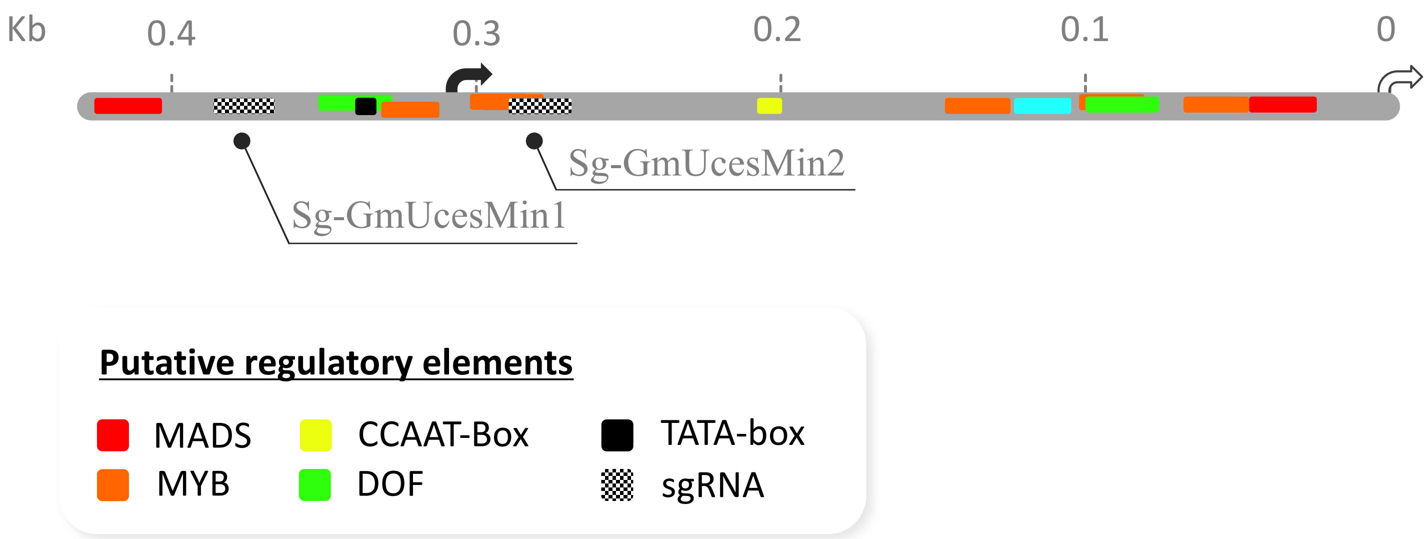
Kb
0.4
0.3
0.2
0.1
0
Sg-GmUcesMin2
Sg-GmUcesMin1
Putative regulatory elements
MADS
CCAAT-Box
TATA-box
MYB
DOF
sgRNA

Figure S3

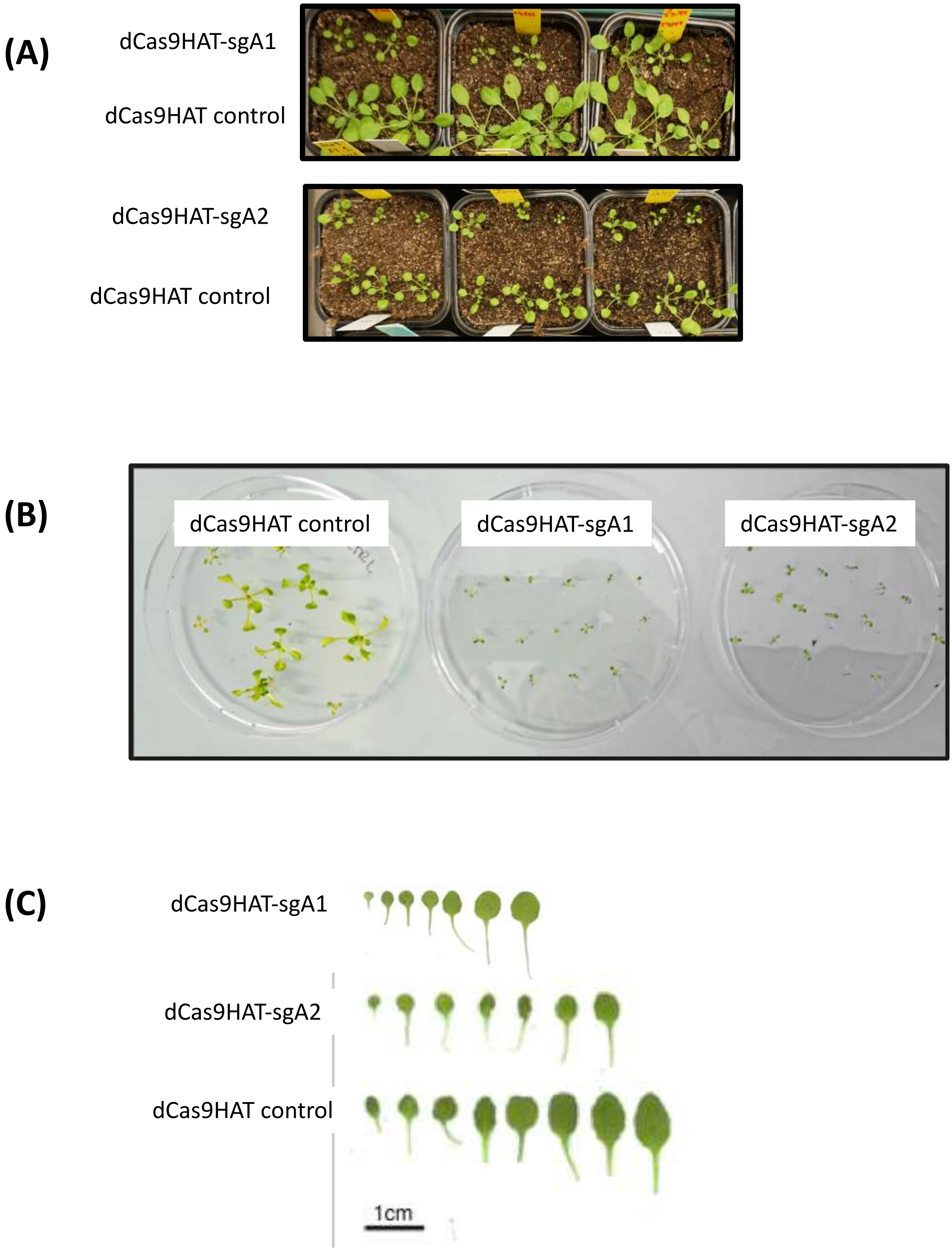

(A)
dCas9HAT-sgA1
dCas9HAT control
dCas9HAT-sgA2
dCas9HAT control
(B)
dCas9HAT control
dCas9HAT-sgA1
dCas9HAT-sgA2
(C)
dCas9HAT-sgA1
dCas9HAT-sgA2
dCas9HAT control
1cm

Figure S4

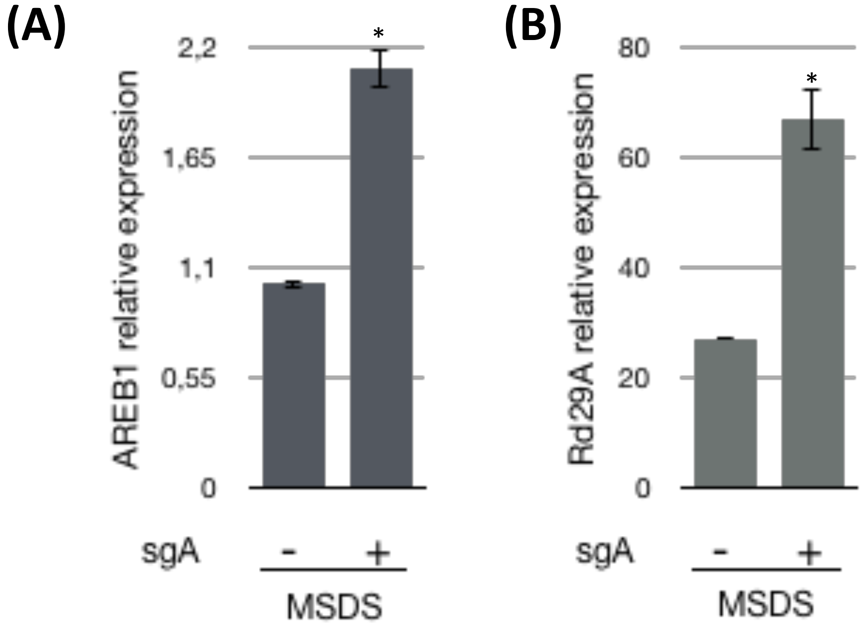

(A)
AREB1 relative expression
2,2
1,65
1,1
0,55
0
*
sgA
-
+
MSDS
(B)
Rd29A relative expression
80
60
40
20
0
*
sgA
-
+
MSDS

Figure S5

dCas9HAT-sgA2 dCas9HAT control

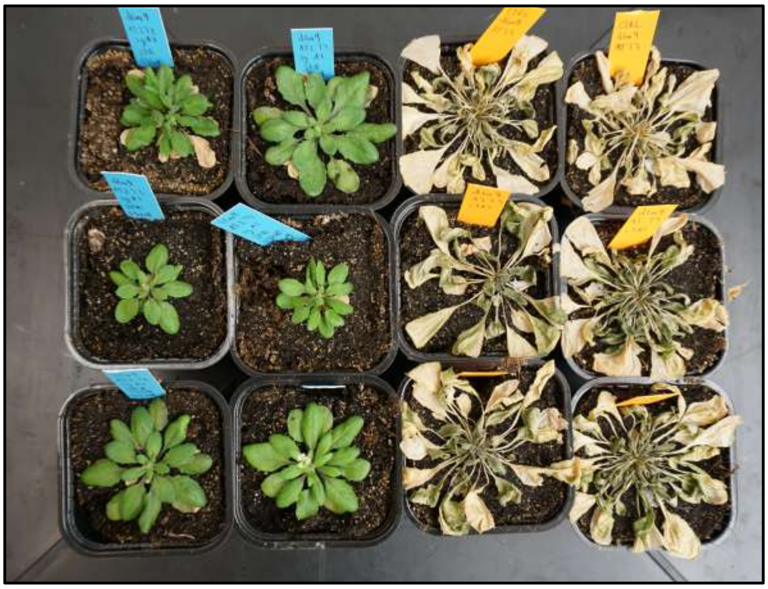

Table S1 - Vectors used in this study

pGreenII-dCas9HAT	TTTTTATCCCCGGAAGCCTGTGGATAGAGGGTAGTTATCCACGTGAAACCGCTAATGCCCCGCAAAGCCTTGATTCACGGGGCTTTCCGGCCCGCTCCAAAAACTATCCACGTGAAATCGCTAATCAGGGTACGTGAAATCGCTAATCGGAGTACGTGAAATCGCTAATAAGGTCACGTGAAATCGCTAATCAAAAAGGCACGTGAGAACGCTAATAGCCCTTTCAGA<br>TCAACAGCTTGCAAACACCCCTCGCTCCGGCAAGTAGTTACAGCAAGTAGTATGTTCAATTAGCTTTTCAATTATGAATATATATATCAATTATTGGTCGCCCTTGGCTTGTGGACAATGCGCTACGCGCACCGGCTCCGCCCGTGGACAACCGCAAGCGGTTGCCCACCGTCGAGCGCCAGCGCCTTTGCCCACAACCCGGCGGCCGGCCGCAACAGATCGTTTTATA<br>AATTTTTTTTTTTGAAAAAGAAAAAGCCCGAAAGGCGGCAACCTCTCGGGCTTCTGGATTTCCGATCCCCGGAATTAGAgatcTTGGCAGGATATATTGTGGTGTAACGTTATCGATCTGGATTTTAGTACTGGATTTTGGTTTTAGGAATTAGAAATTTTATTGATAGAAGTATTTTACAAATACAAATACATACTAAGGGTTTCTTATATGCTCAACACATGAGCGAAA<br>CCCTATAAGAACCCTAATTTCCCTTATCGGGAAACTACTCACACATTATTTATGGAGAAAATAGAGAGAGATAGATTTGTAGAGAGAGACTGGTGATTTCAGCGTACCGAATTAATTCTCCCGCGACCAGCCGAGCGAGCTTAGCGAACTGTGGACGAGAACTGTGCCACCAAGCGTAAGGCCGTTCTCTCGCATTTGCCTTGCTAGGCTCGCGCGAGTTGCTGGCTG<br>AGGCGTTCTCGAAATCAGCTCTTGTTCGGTCGGCATCTACTCTATTCCTTTGCCCTCGGACGAGTGCTGGGGCGTCGGTTTCCACTATCGGCGAGTACTTCTACACAGCCATCGGTCCAGACGGCCGCGCTTCTGCGGGCGATTTGTGTACGCCCGACAGTCCCGGCTCCGGATCGGACGATTGCGTCGCATCGACCCTGCGCCCAAGCTGCATCATCGAAATTGCCGT<br>CAACCAAGCTCTGATAGAGTTGGTCAAGACCAATGCGGAGCATATACGCCCGGAGGCGTGGCGATCCTGCAAGCTCCGGATGCCTCCGCTCGAAGTAGCGCGTCTGCTGCTCCATACAAGCCAACCACGGCCTCCAGAAGAAGATGTTGGCGACCTCGTATTGGGAATCCCCGAACATCGCCTCGCTCCAGTCAATGACCGCTGTTATGCGGCCATTGTCCGTCAGG<br>ACATTGTTGGAGCCGAAATCCGCGTGCACGAGGTGCCGGACTTCGGGGCAGTCCTCGGCCCAAAGCATCAGCTCATCGAGAGCCTGCGCGACGGACGCACTGACGGTGTCGTCCATCACAGTTTGCCAGTGATACACATGGGGATCAGCAATCGCGCATATGAAATCACGCCATGTAGTGTATTGACCGATTCCTTGCGGTCCGAATGGGCCGAACCCGCTCGTCT<br>GGCTAAGATCGGCCGCAGCGATCGCATCCATAGCCTCCGCGACCGGCTGAAGAACAGCGGGCAGTTCGGTTTCAGGCAGGTCTTGCAACGTGACACCCTGTGCACGGCGGGAGATGCAATAGGTCAGGCTCTCGCTGAACTCCCCAATGTCAAGCACTTCCGGAATCGGGAGCGCGGCCGATGCAAAGTGCCGATAAACATAACGATCTTTGTAGAAACCATCGG<br>CGCAGCTATTTACCCGCAGGACATATCCACGCCCTCCTACATCGAAGCTGAAAGCACGAGATTCTTCGCCCTCCGAGAGCTGCATCAGGTCGGAGACGCTGTCGAACTTTTCGATCAGAAACTTCTCGACAGACGTCGCGGTGAGTTCAGGCTTTTTCATAGGGATCAGCTTGGGCTGTCCTCTCCAAATGAAATGAACTTCCTTATATAGAGGAAGGGTCTTGCGAA<br>GGATAGTGGGATTGTGCGTCATCCCTTACGTCAGTGGAGATGTCACATCAATCCACTTGCTTTGAAGACGTGGTTGGAACGTCTTCTTTTTCCACGATGCTCCTCGTGGGTGGGGGTCCATCTTTGGGACCACTGTCGGCAGAGGCATCTTGAATGATAGCCTTTCCTTTATCGCAATGATGGCATTTGTAGGAGCCACCTTCCTTTTCTACTGTCCTTTCGATGAAGTG<br>ACAGATAGCTGGGCAATGGAATCCGAGGAGGTTTCCCGAAATTACCCTTTGTTGAAAAGTCTCAATAGCCCTTTGGTCTTCTGAGACTGTATCTTTGACATTTTTGGAGTAGGGGTACGATAACATTAACGTTTACAATTTCGCGCCATTCGCCATTCAGGCTGCGCAGgatctggattttagtactggattttggttttaggaattagaaattttattgatagaagtattttacaaatacaaatacata<br>ctaagggtttcttatatgctcaacacatgagcgaaaccctataagaaccctaatttcccttatcgggaaactactcacacattatttatggagaaaTTActttgttagcagccggatctcacttAGCGCTCCTCCTAGGGTCAaGCATTCTTGTAAAGCTCATCCATTCCACCAGTAGAATGTCTTCCCTCAGCTCTCTCGTACTGCTCAACGATAGTGTAATCCTCGTTGTGGGAAGTGATGTCGAGCTTGATACCCAC<br>AATGTAAGCGCCTGGGAGCTGAACTGGCTTCTTAGCCTTGTAAGTAGTCTTAACCTCGGAAGTGTAGTGGCCGCCATCCTTAAGTTTGAGCCTCATCTTGATCTCACCCTTGAGAGCACCATCTTCAGGATACATTCTCTCGGAGGAAGCCTCCCATCCCATAGTCTTTTTCTGCATCACTGGGCCATCAGATGGGAAGTTAGTTCCCCTGAGCTTCACCTTGTAGATGAA<br>CTCGCCATCCTGAAGTGAAGAATCCTGAGTCACAGTCACCACACCACCATCCTCGAAATTCATAACCCTCTCCCACTTGAAGCCCTCTGGGAATGAGAGCTTGAAGTAATCTGGGATGTCGGCTGGGTGCTTAACATAAGCCTTGGAGCCGTAAGTGAACTGTGGGGACAAGATGTCCCAAGCGAATGGAAGTGGTCCACCCTTTGTCACCTTGAGCTTAGCAGTCTG<br>AAATCCCTCGTATGGTCTACCCTCACCCTCACCTTCAATCTCGAACTCGTGTCCGTTGACAGAGCCTTCCATCCTCACCTTGAACCTCATGAACTCTTTGATGATAGCCATGTTGTTCTCTTCGCCCTTGGACACCATttGCATTCcTACTTTTCTCTTCTTTTTTGGCATCCTAGGgTCGAGATGTCCTTTGCTGGACTTACCAtgtcctctccaaatgaaatgaacttccttatatagaggaa<br>gggtcttgcgaaggatagtgggattgtgcgtcatcccttacgtcagtggagatgtcacatcaatccacttgctttgaagacgtggttggaacgtcttctttttccacgatgctcctcgtgggtgggggtccatctttgggaccactgtcggcagaggcatcttgaatgatagcctttcctttatcgcaatgatggcatttgtaggagccaccttccttttctactgtcctttcgatgaagtgacagatagctgggcaatggaatccgaggaggtttcccgaaattaccctttgttgaaaagtc<br>tcaTGTAAAACGACGGCCAGTGaccgGACGTGggACTAGTGTCGACTACGTAtgagacttttcaacaaaggataatttcgggaaacctcctcggattccattgcccagctatctgtcacttcatcgaaaggacagtagaaaaggaaggtggctcctacaaatgccatcattgcgataaaggaaaggctatcattcaagatctctctgccgacagtggtcccaaagatggacccccacccacgaggagcatcgtggaaaaagaagacgttccaaccacgtct<br>tcaaagcaagtggattgatgtgacatctccactgacgtaagggatgacgcacaatcccactatccttcgcaagacccttcctctatataaggaagttcatttcatttggagaggacatatttttacaacaattaccaacaacaacaaacaacaaacaacattacaattactatttacaattaccacTTCGAAATGGACAAGAAGTACTCCATTGGGCTCGCTATCGGCACAAACAGCGTCGGCTGGGCCGTCATTACGGACGAGTACAAG<br>GTGCCGAGCAAAAAAATTCAAAGTTCTGGGCAATACCGATCGCCACAGCATAAAGAAGAACCTCATTGGCGCCCTCCTGTTCGACTCCGGGGAGACGGCCGAAGCCACGCGGCTCAAAAGAACAGCACGGCGCAGATATACCCGCAGAAAGAATCGGATCTGCTACCTGCAGGAGATCTTTAGTAATGAGATGGCTAAGGTGGATGACTCTTTCTTCCATAGGCTGG
pGreenII-sgRNA 1	AGCTCTCATCAACCGTGGCTCCCTCACTTTCTGGCTGGATGATGGGGCGATTCAGGCGATCCCCATCCAACAGCCCGCCGTCGAGCGGGCTTTTTTATCCCCGGAAGCCTGTGGATAGAGGGTAGTTATCCACGTGAAACCGCTAATGCCCCGCAAAGCCTTGATTCACGGGGCTTTCCGGCCCGCTCCAAAAACTATCCACGTGAAATCGCTAATCAGGGTACGTGA<br>AATCGCTAATCGGAGTACGTGAAATCGCTAATAAGGTCACGTGAAATCGCTAATCAAAAAGGCACGTGAGAACGCTAATAGCCCTTTCAGATCAACAGCTTGCAAACACCCCTCGCTCCGGCAAGTAGTTACAGCAAGTAGTATGTTCAATTAGCTTTTCAATTATGAATATATATATCAATTATTGGTCGCCCTTGGCTTGTGGACAATGCGCTACGCGCACCGGCTC<br>CGCCCGTGGACAACCGCAAGCGGTTGCCCACCGTCGAGCGCCAGCGCCTTTGCCCACAACCCGGCGGCCGGCCGCAACAGATCGTTTTATAAATTTTTTTTTTTGAAAAAGAAAAAGCCCGAAAGGCGGCAACCTCTCGGGCTTCTGGATTTCCGATCCCCGGAATTAGAGATCTTGGCAGGATATATTGTGGTGTAACGTTATCAGCTTGCATGCCGGTCGATCTAG<br>TAACATAGATGACACCGCGCGCGATAATTTATCCTAGTTTGCGCGCTATATTTTGTTTTCTATCGCGTATTAAATGTATAATTGCGGGACTCTAATCAAAAAACCCATCTCATAAATAACGTCATGCATTACATGTTAATTATTACATGCTTAACGTAATTCAACAGAAATTATATGATAATCATCGCAAGACCGGCAACAGGATTCAATCTTAAGAAACTTTATTGCCAAA<br>TGTTTGAACGATCTGCTTGACTCTAGGGGTCATCAGATTTCGGTGACGGGCAGGACCGGACGGGGCGGCACCGGCAGGCTGAAGTCCAGCTGCCAGAAACCCACGTCATGCCAGTTCCCGTGCTTGAAGCCGGCCGCCCGCAGCATGCCACGGGGGGCATATCCGAGCGCCTCGTGCATGCGCACGCTCGGGTCGTTGGGCAGCCCGATGACAGCGACCACGCTC<br>TTGAAGCCCTGTGCCTCCAGGGACTTCAGCAGGTGGGTGTAGAGCGTGGAGCCCAGTCCCGTCCGCTGGTGGCGGGGGGAGACGTACACGGTTGACTCGGCCGTCCAGTCGTAGGCGTTGCGTGCCTTCCAGGGACCCGCGTAGGCGATGCCGGCGACCTCGCCGTCCACCTCGGCGACGAGCCAGGGATAGCGCTCCCGCAGACGGACGAGGTCGTCCGTCCA<br>CTCCTGCGGTTCCTGCGGCTCGGTACGGAAGTTGACCGTGCTTGTCTGGATGTAGTGGTTGACGATGGTGCAGACCGCCGGCATGTCCGCCTCGGTGGCACGGCGGATGTCGGCCGGGCGTCGTTCTGGGCTCATGGTAGATCCCCCTCGATCGAGTTGAGAGTGAATATGAGACTCTAATTGGATACCGAGGGGAATTTATGGAACGTCAGTGGAGCATTTTTGA<br>CAAGAAATATTTGCTAGCTGATAGTGACCTTAGGCGACTTTTGAACGCGCAATAATGGTTTCTGACGTATGTGCTTAGCTCATTAAACTCCAGAAACCCGGCTGAGTGGCTCCTTCAACGTTGCGGTTCTGTCAGTTCCAAACGTAAAACGGCTTGTCCCGCGTCATCGGCGGGGGTCATAACGTGACTCCCTTAATTCTCATGTATCGATAACATTAACGTTTACAATT<br>TCGCGCCATTCGCCATTCAGGCTGCGCAACTGTTGGGAAGGGCGATCGGTGCGGGCCTCTTCGCTATTACGCCAGCTGGCGAAAGGGGGATGTGCTGCAAGGCGATTAAGTTGGGTAACGCCAGGGTTTTCCCAGTCACGACGTTGTAAAACGACGGCCAGTGAATTGTAATACGACTCACTATAGGGCGAATTGGGTACCGGGCCCCCCCTCGAGGTCGACGGT<br>ATCGATAAGCTTGATATCGAATTCCTGCAGCCCtttcacaccgcagggtaataactgatataattaaattgaagctctaGATATCtcgCCCGGGacgTACGTAAGAAATCTCAAAATTCCGGCAGAACAATTTTGAATCTCGATCCGTAGAAACGAGACGGTCATTGTTTTAGTTCCACCACGATTATATTTGAAATTTACGTGAGTGTGAGTGAGACTTGCATAAGAAAATAAAATCTTTA<br>GTTGGGAAAAAATTCAATAATATAAATGGGCTTGAGAAGGGGTACCAAGCGAGGGATAGGCCTTTTTCTAAAATAGGCCCATTTAAGCTATTAACAATCTTCAAAAGTACCACAGCGCTTAGGTAAAGAAAGCAGCTGAGTTTATATATGGTTAGAGACGAAGTAGTGATTGGCAAAATGTCCCTTTTTGGTGTTTTAGAGCTAGAAATAGCAAGTTAAAATAAGGC<br>TAGTCCGTTATCAACTTGAAAAAGTGGCACCGAGTCGGTGCTTTTTTTGGTCTCTACAGGATCTGACATTATTATTGTTGGAAGAGGACTATTTGCAGGGGGATCCACTAGTTCTAGAGCGGCCGCCACCGCGGTGGAGCTCCAGCTTTTGTTCCCTTTAGTGAGGGTTAATTCCGAGCTTGGCGTAATCATGGTCATAGCTGTTTCCTGTGTGAAATTGTTATCCGCTC<br>ACAATTCCACACAACATACGAGCCGGAAGHCATAAAGTGTAAAGCCTGGGGTGCCTAATGAGTGAGCTAACTCACATTAATTGCGTTGCGCTCACTGCCCGCTTTCCAGTCGGGAAACCTGTCGTGCCAGCTGCATTAATGAATCGGCCAACGCGCGGGGAGAGGCGGTTTGCGTATTGGGCGCTCTTCCGCTTCCTCGCTCACTGACTCGCTGCGCTCGGTCGTTCG<br>GCTGCGGCGAGCGGTATCAGCTCACTCAAAGGCGGTAATACGGTTATCCACAGAATCAGGGGATAACGCAGGAAAGAACATGAAGGCCTTGACAGGATATATTGGCGGGTAAACTAAGTCGCTGTATGTGTTTGTTTGAGATCTCATGTGAGCAAAAGGCCAGCAAAAGGCCAGGAACCGTAAAAAGGCCGCGTTGCTGGCGTTTTTCCATAGGCTCCGCCCCCCT<br>GACGAGCATCACAAAAATCGACGCTCAAGTCAGAGGTGGCGAAACCCGACAGGACTATAAAGATACCAGGCGTTTCCCCCTGGAAGCTCCCTCGTGCGCTCTCCTGTTCCGACCCTGCCGCTTACCGGATACCTGTCCGCCTTTCTCCCTTCGGGAAGCGTGGCGCTTTCTCATAGCTCACGCTGTAGGTATCTCAGTTCGGTGTAGGTCGTTCGCTCCAAGCTGGGCT<br>GTGTGCACGAACCCCCCGTTCAGCCCGACCGCTGCGCCTTATCCGGTAACTATCGTCTTGAGTCCAACCCGGTAAGACACGACTTATCGCCACTGGCAGCAGCCACTGGTAACAGGATTAGCAGAGCGAGGTATGTAGGCGGTGCTACAGAGTTCTTGAAGTGGTGGCCTAACTACGGCTACACTAGAAGAACAGTATTTGGTATCTGCGCTCTGCTGAAGCCAGTT<br>ACCTTCGGAAGAAGAGTTGGTAGCTCTTGATCCGGCAAACAAACCACCGCTGGTAGCGGTGGTTTTTTTGTTTGCAAGCAGCAGATTACGCGCAGAAAAAAAGGATCTCAAGAAGATCCTTTGATCTTTTCTACGGGGTCTGACGCTCAGTGGAACGAAAACTCACGTTAAGGGATTTTGGTCATGAGATTATCAAAAAGGATCTTCACCTAGATCCTTTTAAATTAA<br>AAATGAAGTTTTAAATCAATCTAAAGTATATATGTGTAACATTGGTCTAGTGATTAGAAAAACTCATCGAGCATCAAATGAAACTGCAATTTATTCATATCAGGATTATCAATACCATATTTTTGAAAAAGCCGTTTCTGTAATGAAGGAGAAAACTCACCGAGGCAGTTCCATAGGATGGCAAGATCCTGGTATCGGTCTGCGATTCCGACTCGTCCAACATCAATACA<br>ACCTATTAATTTCCCCTCGTCAAAAATAAGGTTATCAAGTGAGAAATCACCATGAGTGACGACTGAATCCGGTGAGAATGGCAAAAGTTTATGCATTTCTTTCCAGACTTGTTCAACAGGCCAGCCATTACGCTCGTCATCAAAATCACTCGCATCAACCAAACCGTTATTCATTCGTGATTGCGCCTGAGCGAGACGAAATACGCGATCGCTGTTAAAAGGACAATTAC<br>AAACAGGAATCGAATGCAACCGGCGCAGGAACACTGCCAGCGCATCAACAATATTTTCACCTGAATCAGGATATTCTTCTAATACCTGGAATGCTGTTTTCCCTGGGATCGCAGTGGTGAGTAACCATGCATCATCAGGAGTACGGATAAAATGCTTGATGGTCGGAAGAGGCATAAATTCCGTCAGCCAGTTTAGTCTGACCATCTCATCTGTAACAACATTGGCAA<br>CGCTACCTTTGCCATGTTTCAGAAACAACTCTGGCGCATCGGGCTTCCCATACAATCGGTAGATTGTCGCACCTGATTGCCCGACATTATCGCGAGCCCATTTATACCCATATAAATCAGCATCCATGTTGGAATTTAATCGCGGCCTTGAGCAAGACGTTTCCCGTTGAATATGGCTCATAACACCCCTTGTATTACTGTTTATGTAAGCAGACAGTTTTATTGTTCATGA<br>TGATATATTTTTATCTTGTGCAATGTAACATCAGAGATTTTGAGACACAACGTGGCTTTGTTGAATAAATCGAACTTTTGCTGAGTTGAAGGATCAGATCACGCATCTTCCCGACAACGCAGACCGTTCCGTGGCAAAGCAAAAGTTCAAAATCACCAACTGGTCCACCTACAACAA
pGreenII-sgRNA 2	AGCTCTCATCAACCGTGGCTCCCTCACTTTCTGGCTGGATGATGGGGCGATTCAGGCGATCCCCATCCAACAGCCCGCCGTCGAGCGGGCTTTTTTATCCCCGGAAGCCTGTGGATAGAGGGTAGTTATCCACGTGAAACCGCTAATGCCCCGCAAAGCCTTGATTCACGGGGCTTTCCGGCCCGCTCCAAAAACTATCCACGTGAAATCGCTAATCAGGGTACGTGA<br>AATCGCTAATCGGAGTACGTGAAATCGCTAATAAGGTCACGTGAAATCGCTAATCAAAAAGGCACGTGAGAACGCTAATAGCCCTTTCAGATCAACAGCTTGCAAACACCCCTCGCTCCGGCAAGTAGTTACAGCAAGTAGTATGTTCAATTAGCTTTTCAATTATGAATATATATATCAATTATTGGTCGCCCTTGGCTTGTGGACAATGCGCTACGCGCACCGGCTC<br>CGCCCGTGGACAACCGCAAGCGGTTGCCCACCGTCGAGCGCCAGCGCCTTTGCCCACAACCCGGCGGCCGGCCGCAACAGATCGTTTTATAAATTTTTTTTTTTGAAAAAGAAAAAGCCCGAAAGGCGGCAACCTCTCGGGCTTCTGGATTTCCGATCCCCGGAATTAGAGATCTTGGCAGGATATATTGTGGTGTAACGTTATCAGCTTGCATGCCGGTCGATCTAG<br>TAACATAGATGACACCGCGCGCGATAATTTATCCTAGTTTGCGCGCTATATTTTGTTTTCTATCGCGTATTAAATGTATAATTGCGGGACTCTAATCAAAAAACCCATCTCATAAATAACGTCATGCATTACATGTTAATTATTACATGCTTAACGTAATTCAACAGAAATTATATGATAATCATCGCAAGACCGGCAACAGGATTCAATCTTAAGAAACTTTATTGCCAAA<br>TGTTTGAACGATCTGCTTGACTCTAGGGGTCATCAGATTTCGGTGACGGGCAGGACCGGACGGGGCGGCACCGGCAGGCTGAAGTCCAGCTGCCAGAAACCCACGTCATGCCAGTTCCCGTGCTTGAAGCCGGCCGCCCGCAGCATGCCACGGGGGGCATATCCGAGCGCCTCGTGCATGCGCACGCTCGGGTCGTTGGGCAGCCCGATGACAGCGACCACGCTC<br>TTGAAGCCCTGTGCCTCCAGGGACTTCAGCAGGTGGGTGTAGAGCGTGGAGCCCAGTCCCGTCCGCTGGTGGCGGGGGGAGACGTACACGGTTGACTCGGCCGTCCAGTCGTAGGCGTTGCGTGCCTTCCAGGGACCCGCGTAGGCGATGCCGGCGACCTCGCCGTCCACCTCGGCGACGAGCCAGGGATAGCGCTCCCGCAGACGGACGAGGTCGTCCGTCCA<br>CTCCTGCGGTTCCTGCGGCTCGGTACGGAAGTTGACCGTGCTTGTCTGGATGTAGTGGTTGACGATGGTGCAGACCGCCGGCATGTCCGCCTCGGTGGCACGGCGGATGTCGGCCGGGCGTCGTTCTGGGCTCATGGTAGATCCCCCTCGATCGAGTTGAGAGTGAATATGAGACTCTAATTGGATACCGAGGGGAATTTATGGAACGTCAGTGGAGCATTTTTGA<br>CAAGAAATATTTGCTAGCTGATAGTGACCTTAGGCGACTTTTGAACGCGCAATAATGGTTTCTGACGTATGTGCTTAGCTCATTAAACTCCAGAAACCCGGCTGAGTGGCTCCTTCAACGTTGCGGTTCTGTCAGTTCCAAACGTAAAACGGCTTGTCCCGCGTCATCGGCGGGGGTCATAACGTGACTCCCTTAATTCTCATGTATCGATAACATTAACGTTTACAATT<br>TCGCGCCATTCGCCATTCAGGCTGCGCAACTGTTGGGAAGGGCGATCGGTGCGGGCCTCTTCGCTATTACGCCAGCTGGCGAAAGGGGGATGTGCTGCAAGGCGATTAAGTTGGGTAACGCCAGGGTTTTCCCAGTCACGACGTTGTAAAACGACGGCCAGTGAATTGTAATACGACTCACTATAGGGCGAATTGGGTACCGGGCCCCCCCTCGAGGTCGACGGT<br>ATCGATAAGCTTGATATCGAATTCCTGCAGCCCtttcacaccgcagggtaataactgatataattaaattgaagctctaGATATCtcgCCCGGGacgTACGTAAGAAATCTCAAAATTCCGGCAGAACAATTTTGAATCTCGATCCGTAGAAACGAGACGGTCATTGTTTTAGTTCCACCACGATTATATTTGAAATTTACGTGAGTGTGAGTGAGACTTGCATAAGAAAATAAAATCTTTA<br>GTTGGGAAAAAATTCAATAATATAAATGGGCTTGAGAAGGGGTACCAAGCGAGGGATAGGCCTTTTTCTAAAATAGGCCCATTTAAGCTATTAACAATCTTCAAAAGTACCACAGCGCTTAGGTAAAGAAAGCAGCTGAGTTTATATATGGTTAGAGACGAAGTAGTGATTGGATTGATTTAAATCAATTTTGTTTTAGAGCTAGAAATAGCAAGTTAAAATAAGGCT<br>AGTCCGTTATCAACTTGAAAAAGTGGCACCGAGTCGGTGCTTTTTTTGGTCTCTACAGGATCTGACATTATTATTGTTGGAAGAGGACTATTTGCAGGGGGATCCACTAGTTCTAGAGCGGCCGCCACCGCGGTGGAGCTCCAGCTTTTGTTCCCTTTAGTGAGGGTTAATTCCGAGCTTGGCGTAATCATGGTCATAGCTGTTTCCTGTGTGAAATTGTTATCCGCTCA<br>CAATTCCACACAACATACGAGCCGGAAGHCATAAAGTGTAAAGCCTGGGGTGCCTAATGAGTGAGCTAACTCACATTAATTGCGTTGCGCTCACTGCCCGCTTTCCAGTCGGGAAACCTGTCGTGCCAGCTGCATTAATGAATCGGCCAACGCGCGGGGAGAGGCGGTTTGCGTATTGGGCGCTCTTCCGCTTCCTCGCTCACTGACTCGCTGCGCTCGGTCGTTCGG<br>CTGCGGCGAGCGGTATCAGCTCACTCAAAGGCGGTAATACGGTTATCCACAGAATCAGGGGATAACGCAGGAAAGAACATGAAGGCCTTGACAGGATATATTGGCGGGTAAACTAAGTCGCTGTATGTGTTTGTTTGAGATCTCATGTGAGCAAAAGGCCAGCAAAAGGCCAGGAACCGTAAAAAGGCCGCGTTGCTGGCGTTTTTCCATAGGCTCCGCCCCCCTG<br>ACGAGCATCACAAAAATCGACGCTCAAGTCAGAGGTGGCGAAACCCGACAGGACTATAAAGATACCAGGCGTTTCCCCCTGGAAGCTCCCTCGTGCGCTCTCCTGTTCCGACCCTGCCGCTTACCGGATACCTGTCCGCCTTTCTCCCTTCGGGAAGCGTGGCGCTTTCTCATAGCTCACGCTGTAGGTATCTCAGTTCGGTGTAGGTCGTTCGCTCCAAGCTGGGCTG<br>TGTGCACGAACCCCCCGTTCAGCCCGACCGCTGCGCCTTATCCGGTAACTATCGTCTTGAGTCCAACCCGGTAAGACACGACTTATCGCCACTGGCAGCAGCCACTGGTAACAGGATTAGCAGAGCGAGGTATGTAGGCGGTGCTACAGAGTTCTTGAAGTGGTGGCCTAACTACGGCTACACTAGAAGAACAGTATTTGGTATCTGCGCTCTGCTGAAGCCAGTTA<br>CCTTCGGAAGAAGAGTTGGTAGCTCTTGATCCGGCAAACAAACCACCGCTGGTAGCGGTGGTTTTTTTGTTTGCAAGCAGCAGATTACGCGCAGAAAAAAAGGATCTCAAGAAGATCCTTTGATCTTTTCTACGGGGTCTGACGCTCAGTGGAACGAAAACTCACGTTAAGGGATTTTGGTCATGAGATTATCAAAAAGGATCTTCACCTAGATCCTTTTAAATTAAA<br>AATGAAGTTTTAAATCAATCTAAAGTATATATGTGTAACATTGGTCTAGTGATTAGAAAAACTCATCGAGCATCAAATGAAACTGCAATTTATTCATATCAGGATTATCAATACCATATTTTTGAAAAAGCCGTTTCTGTAATGAAGGAGAAAACTCACCGAGGCAGTTCCATAGGATGGCAAGATCCTGGTATCGGTCTGCGATTCCGACTCGTCCAACATCAATACAA<br>CCTATTAATTTCCCCTCGTCAAAAATAAGGTTATCAAGTGAGAAATCACCATGAGTGACGACTGAATCCGGTGAGAATGGCAAAAGTTTATGCATTTCTTTCCAGACTTGTTCAACAGGCCAGCCATTACGCTCGTCATCAAAATCACTCGCATCAACCAAACCGTTATTCATTCGTGATTGCGCCTGAGCGAGACGAAATACGCGATCGCTGTTAAAAGGACAATTACA<br>AACAGGAATCGAATGCAACCGGCGCAGGAACACTGCCAGCGCATCAACAATATTTTCACCTGAATCAGGATATTCTTCTAATACCTGGAATGCTGTTTTCCCTGGGATCGCAGTGGTGAGTAACCATGCATCATCAGGAGTACGGATAAAATGCTTGATGGTCGGAAGAGGCATAAATTCCGTCAGCCAGTTTAGTCTGACCATCTCATCTGTAACAACATTGGCAAC<br>GCTACCTTTGCCATGTTTCAGAAACAACTCTGGCGCATCGGGCTTCCCATACAATCGGTAGATTGTCGCACCTGATTGCCCGACATTATCGCGAGCCCATTTATACCCATATAAATCAGCATCCATGTTGGAATTTAATCGCGGCCTTGAGCAAGACGTTTCCCGTTGAATATGGCTCATAACACCCCTTGTATTACTGTTTATGTAAGCAGACAGTTTTATTGTTCATGAT<br>GATATATTTTTATCTTGTGCAATGTAACATCAGAGATTTTGAGACACAACGTGGCTTTGTTGAATAAATCGAACTTTTGCTGAGTTGAAGGATCAGATCACGCATCTTCCCGACAACGCAGACCGTTCCGTGGCAAAGCAAAAGTTCAAAATCACCAACTGGTCCACCTACAACAA
pGreenII-sgRNAAREB1	AGCTCTCATCAACCGTGGCTCCCTCACTTTCTGGCTGGATGATGGGGCGATTCAGGCGATCCCCATCCAACAGCCCGCCGTCGAGCGGGCTTTTTTATCCCCGGAAGCCTGTGGATAGAGGGTAGTTATCCACGTGAAACCGCTAATGCCCCGCAAAGCCTTGATTCACGGGGCTTTCCGGCCCGCTCCAAAAACTATCCACGTGAAATCGCTAATCAGGGTACGTGA<br>AATCGCTAATCGGAGTACGTGAAATCGCTAATAAGGTCACGTGAAATCGCTAATCAAAAAGGCACGTGAGAACGCTAATAGCCCTTTCAGATCAACAGCTTGCAAACACCCCTCGCTCCGGCAAGTAGTTACAGCAAGTAGTATGTTCAATTAGCTTTTCAATTATGAATATATATATCAATTATTGGTCGCCCTTGGCTTGTGGACAATGCGCTACGCGCACCGGCTC<br>CGCCCGTGGACAACCGCAAGCGGTTGCCCACCGTCGAGCGCCAGCGCCTTTGCCCACAACCCGGCGGCCGGCCGCAACAGATCGTTTTATAAATTTTTTTTTTTGAAAAAGAAAAAGCCCGAAAGGCGGCAACCTCTCGGGCTTCTGGATTTCCGATCCCCGGAATTAGAGATCTTGGCAGGATATATTGTGGTGTAACGTTATCAGCTTGCATGCCGGTCGATCTAG<br>TAACATAGATGACACCGCGCGCGATAATTTATCCTAGTTTGCGCGCTATATTTTGTTTTCTATCGCGTATTAAATGTATAATTGCGGGACTCTAATCAAAAAACCCATCTCATAAATAACGTCATGCATTACATGTTAATTATTACATGCTTAACGTAATTCAACAGAAATTATATGATAATCATCGCAAGACCGGCAACAGGATTCAATCTTAAGAAACTTTATTGCCAAA<br>TGTTTGAACGATCTGCTTGACTCTAGGGGTCATCAGATTTCGGTGACGGGCAGGACCGGACGGGGCGGCACCGGCAGGCTGAAGTCCAGCTGCCAGAAACCCACGTCATGCCAGTTCCCGTGCTTGAAGCCGGCCGCCCGCAGCATGCCACGGGGGGCATATCCGAGCGCCTCGTGCATGCGCACGCTCGGGTCGTTGGGCAGCCCGATGACAGCGACCACGCTC<br>TTGAAGCCCTGTGCCTCCAGGGACTTCAGCAGGTGGGTGTAGAGCGTGGAGCCCAGTCCCGTCCGCTGGTGGCGGGGGGAGACGTACACGGTTGACTCGGCCGTCCAGTCGTAGGCGTTGCGTGCCTTCCAGGGACCCGCGTAGGCGATGCCGGCGACCTCGCCGTCCACCTCGGCGACGAGCCAGGGATAGCGCTCCCGCAGACGGACGAGGTCGTCCGTCCA<br>CTCCTGCGGTTCCTGCGGCTCGGTACGGAAGTTGACCGTGCTTGTCTGGATGTAGTGGTTGACGATGGTGCAGACCGCCGGCATGTCCGCCTCGGTGGCACGGCGGATGTCGGCCGGGCGTCGTTCTGGGCTCATGGTAGATCCCCCTCGATCGAGTTGAGAGTGAATATGAGACTCTAATTGGATACCGAGGGGAATTTATGGAACGTCAGTGGAGCATTTTTGA<br>CAAGAAATATTTGCTAGCTGATAGTGACCTTAGGCGACTTTTGAACGCGCAATAATGGTTTCTGACGTATGTGCTTAGCTCATTAAACTCCAGAAACCCGGCTGAGTGGCTCCTTCAACGTTGCGGTTCTGTCAGTTCCAAACGTAAAACGGCTTGTCCCGCGTCATCGGCGGGGGTCATAACGTGATGTAAAACGACGGCCAGTGgctcgtaatgtctgcccattctgctattctg<br>tatacacccgcagagtactgcaatttgactgggtatttcacaccgcagggtaataactgatataattaaattgaagctctaGATATCtcgCCCGGGacgTACGTAAGAAATCTCAAAATTCCGGCAGAACAATTTTGAATCTCGATCCGTAGAAACGAGACGGTCATTGTTTTAGTTCCACCACGATTATATTTGAAATTTACGTGAGTGTGAGTGAGACTTGCATAAGAAAATAAAATCTTTAGTTGG<br>GAAAAAATTCAATAATATAAATGGGCTTGAGAAGGGGTACCAAGCGAGGGATAGGCCTTTTTCTAAAATAGGCCCATTTAAGCTATTAACAATCTTCAAAAGTACCACAGCGCTTAGGTAAAGAAAGCAGCTGAGTTTATATATGGTTAGAGACGAAGTAGTGATTGGTTCAGATCCAGTTATTAGGGTTTTAGAGCTAGAAATAGCAAGTTAAAATAAGGCTAGTC<br>CGTTATCAACTTGAAAAAGTGGCACCGAGTCGGTGCTTTTTTTGGTCTCTACAGGATCTGACATTATTATTGTTGGAAGAGGACTATTTGCAAAggatccccatttgtcgctcttcgcaatgtcaacagtacccttagtatattctccagtagAGAAATCTCAAAATTCCGGCAGAACAATTTTGAATCTCGATCCGTAGAAACGAGACGGTCATTGTTTTAGTTCCACCACGATTATATTTGAA<br>ATTTACGTGAGTGTGAGTGAGACTTGCATAAGAAAATAAAATCTTTAGTTGGGAAAAAATTCAATAATATAAATGGGCTTGAGAAGGGGTACCAAGCGAGGGATAGGCCTTTTTCTAAAATAGGCCCATTTAAGCTATTAACAATCTTCAAAAGTACCACAGCGCTTAGGTAAAGAAAGCAGCTGAGTTTATATATGGTTAGAGACGAAGTAGTGATTGGGATTTGT<br>CCAAGCAACATTGTTTTAGAGCTAGAAATAGCAAGTTAAAATAAGGCTAGTCCGTTATCAACTTGAAAAAGTGGCACCGAGTCGGTGCTTTTTTTTACGTAAccaGGCGCCaaGCGGGTTAACgcggccgctaatcaaccaatcgtaaccttcatctcttccacccatgtctctttgagcatggagggcacagttaagccgctaaaggcattatccgccaagtacaatttttttactcttcgGTCATAGCTGTTTCCTG<br>ACGCAGGAAAGAACATGAAGGCCTTGACAGGATATATTGGCGGGTAAACTAAGTCGCTGTATGTGTTTGTTTGAGATCTCATGTGAGCAAAAGGCCAGCAAAAGGCCAGGAACCGTAAAAAGGCCGCGTTGCTGGCGTTTTTCCATAGGCTCCGCCCCCCTGACGAGCATCACAAAAATCGACGCTCAAGTCAGAGGTGGCGAAACCCGACAGGACTATAAAGAT<br>ACCAGGCGTTTCCCCCTGGAAGCTCCCTCGTGCGCTCTCCTGTTCCGACCCTGCCGCTTACCGGATACCTGTCCGCCTTTCTCCCTTCGGGAAGCGTGGCGCTTTCTCATAGCTCACGCTGTAGGTATCTCAGTTCGGTGTAGGTCGTTCGCTCCAAGCTGGGCTGTGTGCACGAACCCCCCGTTCAGCCCGACCGCTGCGCCTTATCCGGTAACTATCGTCTTGAGTCCA<br>ACCCGGTAAGACACGACTTATCGCCACTGGCAGCAGCCACTGGTAACAGGATTAGCAGAGCGAGGTATGTAGGCGGTGCTACAGAGTTCTTGAAGTGGTGGCCTAACTACGGCTACACTAGAAGAACAGTATTTGGTATCTGCGCTCTGCTGAAGCCAGTTACCTTCGGAAGAAGAGTTGGTAGCTCTTGATCCGGCAAACAAACCACCGCTGGTAGCGGTGGTTT<br>TTTTGTTTGCAAGCAGCAGATTACGCGCAGAAAAAAAGGATCTCAAGAAGATCCTTTGATCTTTTCTACGGGGTCTGACGCTCAGTGGAACGAAAACTCACGTTAAGGGATTTTGGTCATGAGATTATCAAAAAGGATCTTCACCTAGATCCTTTTAAATTAAAAATGAAGTTTTAAATCAATCTAAAGTATATATGTGTAACATTGGTCTAGTGATTAGAAAAACTCAT<br>CGAGCATCAAATGAAACTGCAATTTATTCATATCAGGATTATCAATACCATATTTTTGAAAAAGCCGTTTCTGTAATGAAGGAGAAAACTCACCGAGGCAGTTCCATAGGATGGCAAGATCCTGGTATCGGTCTGCGATTCCGACTCGTCCAACATCAATACAACCTATTAATTTCCCCTCGTCAAAAATAAGGTTATCAAGTGAGAAATCACCATGAGTGACGACTGA<br>ATCCGGTGAGAATGGCAAAAGTTTATGCATTTCTTTCCAGACTTGTTCAACAGGCCAGCCATTACGCTCGTCATCAAAATCACTCGCATCAACCAAACCGTTATTCATTCGTGATTGCGCCTGAGCGAGACGAAATACGCGATCGCTGTTAAAAGGACAATTACAAACAGGAATCGAATGCAACCGGCGCAGGAACACTGCCAGCGCATCAACAATATTTTCACCTGAA<br>TCAGGATATTCTTCTAATACCTGGAATGCTGTTTTCCCTGGGATCGCAGTGGTGAGTAACCATGCATCATCAGGAGTACGGATAAAATGCTTGATGGTCGGAAGAGGCATAAATTCCGTCAGCCAGTTTAGTCTGACCATCTCATCTGTAACAACATTGGCAACGCTACCTTTGCCATGTTTCAGAAACAACTCTGGCGCATCGGGCTTCCCATACAATCGGTAGATTGT<br>CGCACCTGATTGCCCGACATTATCGCGAGCCCATTTATACCCATATAAATCAGCATCCATGTTGGAATTTAATCGCGGCCTTGAGCAAGACGTTTCCCGTTGAATATGGCTCATAACACCCCTTGTATTACTGTTTATGTAAGCAGACAGTTTTATTGTTCATGATGATATATTTTTATCTTGTGCAATGTAACATCAGAGATTTTGAGACACAACGTGGCTTTGTTGAATA<br>AATCGAACTTTTGCTGAGTTGAAGGATCAGATCACGCATCTTCCCGACAACGCAGACCGTTCCGTGGCAAAGCAAAAGTTCAAAATCACCAACTGGTCCACCTACAACAA

Table S2. sgRNAs used during this study

sgRNA	Target name	Target sequence	PAM	Location (from TSS)
sgRNA 1	GmUcesMin	GATTGATTTAAATCAATTTT	AGG	-56 bp
sgRNA 2	GmUcesMin	GCAAAATGTCCCTTTTTGGT	TGG	+18 bp
sgRNA-AREB1_1	AREB1 promoter	GTTCAGATCCAGTTATTAGG	TGG	-479 bp
sgRNA-AREB1_2	AREB1 promoter	GGATTTGTCCAAGCAACATT	TGG	+356 bp

Table S3. Primers sequences used in this study

Name	Sequence	Use
CAS9 FWRD	GAAAAGGAACAGCGACAAGC	Insertion confirmation
HAC REV	CCTTAAGAGGAGGACAAGCCC	Insertion confirmation
HAC FWRD	TTCCTACTGCTGAATCTCTTGTT	Insertion confirmation
sgRNA1 GBC36 FWRD	TTT CAC ACC GCA GGG TAA TAA CTG	sgRNA synthesis and ampification
sgRNA1 GBD23 REV	TGCAAATAGTCCTCTTCCAACAA	sgRNA synthesis and ampification
sgRNA2 GBE24 FWRD	tttgtcgctcttcgcaatgtc	sgRNA synthesis and ampification
sgRNA2 GBA26 REV	tgctcaaagagacatgggtggaag	sgRNA synthesis and ampification
sgRNA 1 FWD	GATTGATTTAAATCAATTTTGTTTTAGAGCTAGAAATAGCAA	sgRNA synthesis
sgRNA1 REV	AAAATTGATTTAAATCAATCCAATCACTACTTCGTCT	sgRNA synthesis
sgRNA 2 FWD	GCAAAATGTCCCTTTTTGGTGTTTTAGAGCTAGAAATAGCAA	sgRNA synthesis
sgRNA 2 REV	ACCAAAAAGGGACATTTTGCCAATCACTACTTCGTCT	sgRNA synthesis
AREB1_qPCR_2017_F	a a c a g g c t t a c a c c g t g g a g	qPCR
AREB1_qPCR_2017_R	c t t t g g a c c t c c t t g c a g a a	qPCR
dCas9_qPCR_F	AAAGCTCAAAGGGTCTCCCG	qPCR
dCas9_qPCR_R	TTATCGAGGTTAGCGTCGGC	qPCR
Rd29A_qPCR_FWRD	TGGATCTGAAGAACGAATCTGATATC	qPCR
Rd29A_qPCR_REV	GGTCTTCCCTTCGCCAGAA	qPCR
GAPDH A.thaliana_Fow_qPCR	TTGGTGACAACAGGTCAAGCA	qPCR
GAPDH A.thaliana_Rev_qPCR	AAACTTGTCGCTCAATGCAATC	qPCR
ACT2 A.thaliana_Fow_qPCR	CTTGCACCAAGCAGCATGAA	qPCR
ACT2 A.thaliana_Rev_qPCR	CCGATCCAGACACTGTACTTCCTT	qPCR
